# Luminescent Polynuclear Zn- and Cd-Ln Square-Like Nanoclusters With a Flexible Long-Chain Schiff Base Ligand

**DOI:** 10.3389/fchem.2018.00321

**Published:** 2018-07-31

**Authors:** Ting Zhu, Xiaoping Yang, Xiaohui Zheng, Shiqing Wang, Le Bo, Chengri Wang, Hongfen Chen, Dongmei Jiang, Desmond Schipper

**Affiliations:** ^1^Department of Chemistry, College of Chemistry and Materials Engineering, Wenzhou University, Wenzhou, China; ^2^Chemical Biology Research Center, School of Pharmaceutical Science, Wenzhou Medical University, Wenzhou, China; ^3^Department of Chemistry and Biochemistry, The University of Texas at Austin, Austin, TX, United States

**Keywords:** self-assembly, lanthanide, flexible long-chain schiff base ligand, nanoclusters, visible and NIR luminescent

## Abstract

Two series of Zn-Ln and Cd-Ln nanoclusters [Ln_4_Zn_8_L_2_(OAc)_20_(OH)_4_] [Ln = Nd (**1**), Yb (**2**), and Sm (**3**)] and [Ln_2_Cd_2_L_2_(OAc)_2_(OH)_2_(OCH_3_)_2_] [Ln = Nd (**4**), Yb (**5**), and Sm (**6**)] were prepared using a long-chain Schiff base ligand with a flexible (CH_2_)_2_O(CH_2_)_2_O(CH_2_)_2_ chain. All these clusters show square-like structures. The Schiff base ligands show “linear” configurations in the structures of **1**-**6**, and the metric dimensions of Zn-Ln and Cd-Ln clusters measure ~8 × 14 × 21 and 8 × 12 × 12 Å, respectively. The study of luminescence properties shows that the Zn/L and Cd/L chromophores can effectively transfer energy to the lanthanide ions, and **1**-**6** show visible and NIR emissions.

## Introduction

The polynuclear d-f nanoclusters may exhibit specific physical and chemical properties due to the interaction between metal ions (Kauffman et al., 2014; Li et al., 2015). Lanthanide ions have abundant electronic energy levels and can show long-lived and line-like emission bands because of their unique 4f electronic configurations. For example, Nd(III), Er(III), and Yb(III) complexes can show near-infrared (NIR) emissions around 900–1,600 nm, where the absorption of the biological systems and fiber media is low. Consequently, these lanthanide complexes have potential applications in bioassays and luminescent probes (Hemmila and Webb, 1997). In addition, polynuclear d-f nanoclusters with well-defined structures and interesting properties have emerged as a new class of nanomaterials for their potential applications in optoelectronics, magnetism, and as porous materials (Peng et al., 2012; Wang et al., 2013).

**Scheme 1:**
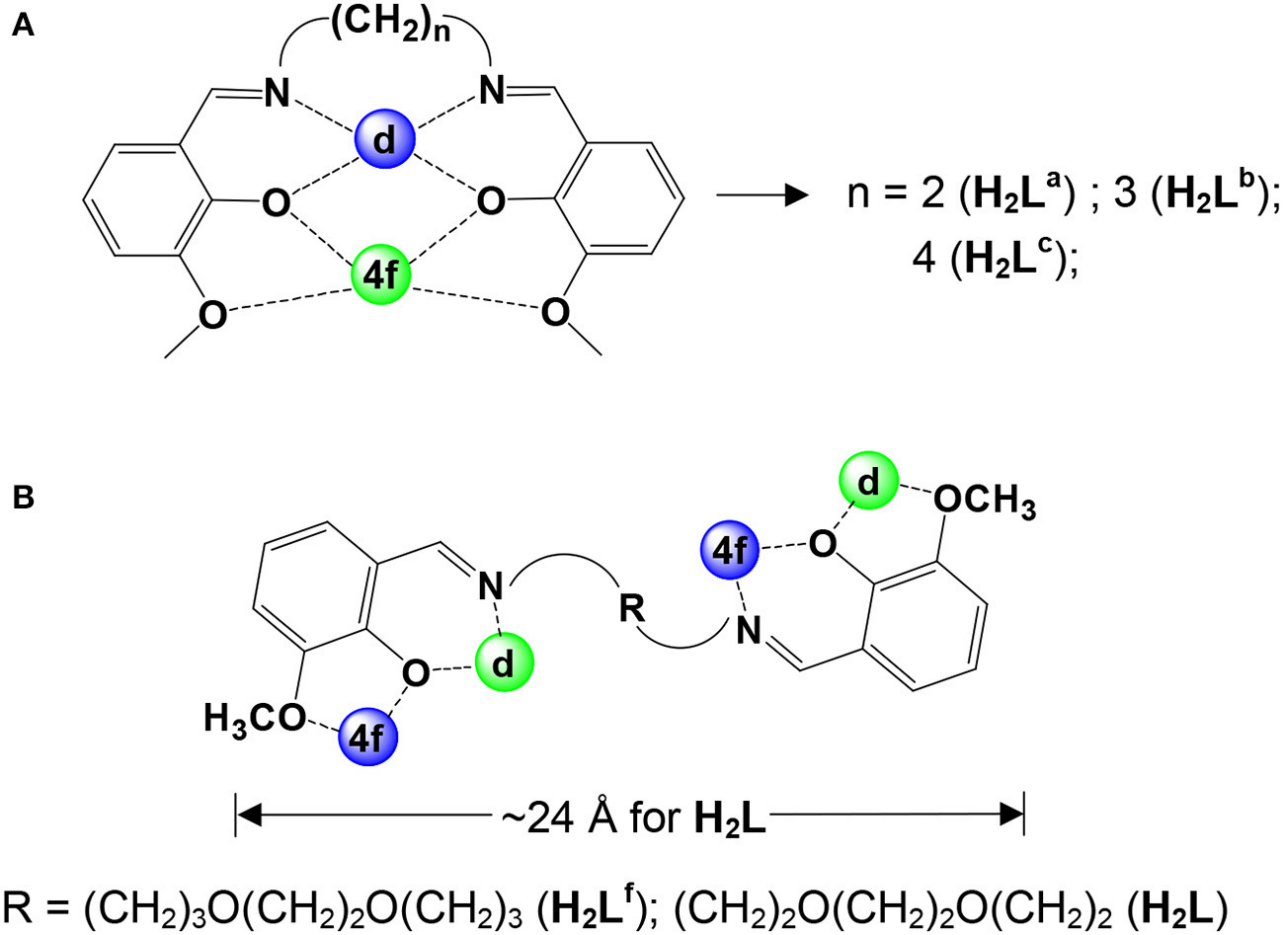
**(A)**“Bending” configuration of H_2_L^a−e^; **(B)**“Linear” configuration of H_2_L^f^ and H_2_L.

Salen-type Schiff base ligands have been widely used to synthesize d-f heteronuclear clusters (Yamaguchi et al., 2010; Pasatoiu et al., 2011, 2012; Watanabe et al., 2011). For example, some polynuclear 3d-4f complexes (3d = Ni, Zn, and Cu) have been synthesized in our group using Schiff base ligands H_2_L^a−c^ that have flexible carbon-carbon backbones (Scheme 1) (Yang et al., 2014). In these polynuclear clusters, the d-metal ions are bound in the N_2_O_2_ cavities and the f-metal ions in the O_2_O_2_ cavities, resulting the classical “bending” configurations of the Schiff base ligands (Scheme 1A). Recently, two kinds of luminescent 24- and 32-metal Cd-Ln complexes were constructed in our studies from Schiff base ligands H_2_L^d^ and H_2_L^e^ (Scheme 1), which have 6 and 8 carbon backbones, respectively (Yang et al., 2013). We have found that the backbone structures of these ligands may affect their coordination modes with metal ions. For example, two 12-metal Zn-Ln nanoclusters [Zn_8_Ln_4_(L^f^)_8_(OAc)_8_](OH)_4_ (Ln = Sm and Nd) were prepared using a long-chain Schiff base ligand with a flexible (CH_2_)_3_O(CH_2_)_2_O(CH_2_)_3_ backbone (Scheme 1B) (Bo et al., 2018). Zinc (II) and Cadmium(II) moieties have been used as efficient energy donors for the luminescence of the Ln(III) ions in Zn- and Cd-Ln complexes (Zheng et al., 2004; Zhu et al., 2006). As part of our continuing studies focused on the studies of luminescent lanthanide-based frameworks, we report here the synthesis and luminescence properties of two series of Zn-Ln and Cd-Ln clusters [Ln_4_Zn_8_L_2_(OAc)_20_(OH)_4_] [Ln = Nd (**1**), Yb (**2**), and Sm (**3**)] and [Ln_2_Cd_2_L_2_(OAc)_2_(OH)_2_(OCH_3_)_2_] [Ln = Nd (**4**), Yb (**5**), and Sm (**6**)] with a long-chain Schiff base ligand N,N'-bis(3-methoxysalicylidene)(1,2-bis(ethoxy)ethane)-1,6-diamine (H_2_L, **Scheme 1B**). The Schiff base ligand H_2_L has a flexible long-chain (CH_2_)_2_O(CH_2_)_2_O(CH_2_)_2_ backbone with two introduced oxygen atoms. Although the long-chain Schiff base ligand H_2_L only has two fewer -CH_2_- groups in the backbone than H_2_L^f^ (Scheme 1B), **1**-**3** show different structures from [Zn_8_Ln_4_(L^f^)_8_(OAc)_8_](OH)_4_ (Ln = Sm and Nd) that have eight Schiff base ligands H_2_L^f^ (Bo et al., 2018). Meanwhile, differing from those clusters with H_2_L^a−e^, all **1**-**6** show interesting square-like structures. The backbone length of H_2_L is ~24 Å, which is much longer than H_2_L^a−c^. It is noticeable that the H_2_L ligand exhibits a different “linear” configuration in **1**-**6** (**Scheme 1B**). Thus, it turns to form large metal clusters. For example, the molecular dimensions of Zn-Ln clusters **1-3** measure ~8 × 14 × 21 Å. The study of luminescence properties shows that all of these clusters display the visible and NIR emissions of lanthanide ions.

## Experimental section

### Preparation of [Nd_4_Zn_8_L_2_(OAc)_20_(OH)_4_] (1)

Zn(OAc)_2_·2H_2_O (0.40 mmol, 0.0876 g), Nd(OAc)_3_·4H_2_O (0.20 mmol, 0.0778 g), and H_2_L (0.05 mmol, 0.0208 g) were dissolved in 12 mL EtOH at room temperature, and a solution of Et_3_N in MeOH (0.01 mol/L, 2 mL) was then added. The mixture was stirred and heated for 30 min under reflux and then filtered. The yellow crystals of **1** were obtained by the diffusion of diethyl ether into the filtrate after 3 weeks. Yield (based on Zn(OAc)_2_·2H_2_O): 0.0937 g (76%). m. p. > 175°C (dec.). EA: C, 31.98; H, 3.97; N, 1.65% (found). Calc. for C_88_H_128_N_4_Nd_4_O_60_Zn_8_, C, 32.01; H, 3.91; N, 1.70%. IR (cm^−1^): 1619 (w), 1557 (m), 1445 (m), 1408 (m), 1334 (w), 1303 (m), 1216 (s), 1086 (s), 1011 (s), 968 (m), 943 (s), 856 (s), 738 (m), 670 (m) (Figure S1).

### Preparation of [Yb_4_Zn_8_L_2_(OAc)_20_(OH)_4_] (2)

The procedure was the same as that for **1** using Yb(OAc)_3_·4H_2_O (0.20 mmol, 0.0842 g). Yellow crystals of **2** were formed after 3 weeks. Yield (based on Zn(OAc)_2_·2H_2_O): 0.0671 g (53%). m. p. > 176°C (dec.). EA: C, 30.83; H, 3.93; N, 1.63% (found). Calc. for C_88_H_128_N_4_O_60_Zn_8_Yb_4_: C, 30.93; H, 3.78; N, 1.64%. IR (CH_3_CN, cm^−1^): 1632 (w), 1557 (w), 1452 (m), 1421 (s), 1291 (m), 1241 (s), 1216 (w), 1080 (s), 1024 (s), 974 (w), 856 (w), 738 (m), 682 (s) (Figure S1).

### Preparation of [Sm_4_Zn_8_L_2_(OAc)_20_(OH)_4_] (3)

The procedure was the same as that for **1** using Sm(OAc)_3_·4H_2_O (0.20 mmol, 0.0803 g). Yellow crystals of **3** were formed after 1 week. Yield (based on Zn(OAc)_2_·2H_2_O): 0.0745 g (60%). m. p. > 188°C (dec.). EA: C, 31.87; H, 3.95; N, 1.59% (found). Calc. for C_88_H_128_N_4_O_60_Zn_8_Sm_4_: C, 31.77; H, 3.88; N, 1.68%. IR (CH_3_CN, cm^−1^): 1632 (w), 1545 (w), 1452 (m), 1421 (s), 1303 (m), 1241 (s), 1216 (w), 1086 (s), 1030 (s), 974 (w), 937 (w), 862 (m), 744 (s), 676 (s) (Figure S1).

### Preparation of [Nd_2_Cd_2_L_2_(OAc)_2_(OH)_2_(OCH_3_)_2_] (4)

Cd(OAc)_2_·2H_2_O (0.20 mmol, 0.0534 g), Nd(NO_3_)_3_·6H_2_O (0.20 mmol, 0.0885 g) and H_2_L (0.20 mmol, 0.0833 g) were dissolved in 12 mL EtOH and 5 mL DMF at room temperature, and a solution of Et_3_N in MeOH (0.01 mol/L, 2 mL) was then added. The mixture was stirred and heated for 30 min under reflux and then filtered. The yellow crystals of **4** were obtained by the diffusion of diethyl ether into the filtrate after 1 week. Yield (based on Nd(NO_3_)_3_·6H_2_O): 0.1238 g (52%). m. p. > 167°C (dec.). EA: C, 38.31; H, 4.41; N, 3.42% (found). Calc. for C_50_H_66_N_4_O_20_Cd_2_Nd_2_: C, 38.59; H, 4.27; N, 3.60%. ESI-MS (CH_3_CN) *m*/*z*: 1558 [M+H]^+^. IR (cm^−1^): 1619 (w), 1545 (m), 1452 (s), 1408 (m), 1303 (m), 1241 (w), 1216 (m), 1080 (s), 1018 (s), 943 (s), 856 (m), 738 (s), 664 (s) (Figures S1 and S2).

### Preparation of [Yb_2_Cd_2_L_2_(OAc)_2_(OH)_2_(OCH_3_)_2_] (5)

The procedure was the same as that for **1** using Yb(NO_3_)_3_·6H_2_O (0.20 mmol, 0.0907 g). Yellow crystals of **5** were formed after 1 week. Yield (based on Yb(NO_3_)_3_·6H_2_O): 0.1449 g (60%). m. p. > 161°C (dec.). EA: C, 36.99; H, 4.38; N, 3.21% (found). Calc. for C_50_H_66_N_4_O_20_Cd_2_Yb_2_: C, 37.21; H, 4.12; N, 3.47%. ESI-MS (CH_3_CN) *m*/*z*: 1597 [M-OH]^+^. IR (CH_3_CN, cm^−1^): 1632 (w), 1551 (m), 1445 (s), 1402 (m), 1340 (m), 1296 (w), 1241 (m), 1222 (s), 1086 (s), 1018 (s), 968 (m), 850 (s), 782 (s), 738 (s), 695 (s) (Figures S1 and S2).

### Preparation of [Sm_2_Cd_2_L_2_(OAc)_2_(OH)_2_(OCH_3_)_2_] (6)

The procedure was the same as that for **1** using Sm(NO_3_)_3_·6H_2_O (0.20 mmol, 0.0903 g). Yellow crystals of **6** were formed after 1 week. Yield (based on Sm(NO_3_)_3_·6H_2_O): 0.1593 g (68%). m. p. > 190°C (dec.). EA: C, 38.17; H, 4.53; N, 3.26% (found). Calc. for C_50_H_66_N_4_O_20_Cd_2_Sm_2_: C, 38.28; H, 4.24; N, 3.57%. IR (CH_3_CN, cm^−1^): 1619 (w), 1545 (m), 1452 (s), 1408 (m), 1303 (m), 1247 (w), 1216 (m), 1080 (s), 1024 (s), 968 (s), 937 (s), 856 (m), 744 (s), 676 (s) (Figure S1).

## Results and discussion

### Synthesis and crystal structures of the clusters

**Figure 1:**
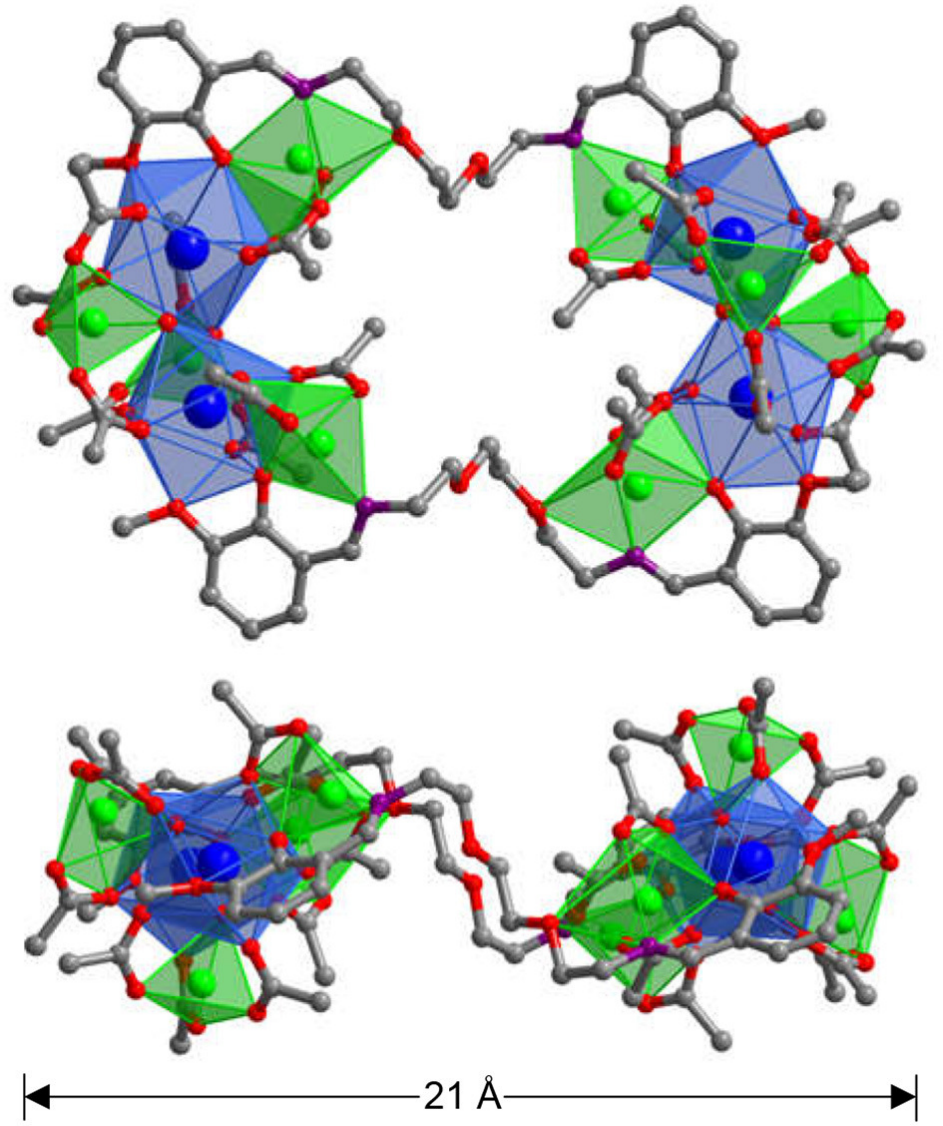
The square-like crystal structure of **1** (**Top**: viewed along the *a*-axis; **Lower**: viewed along the *b*-axis. The color of Nd^3+^ and Zn^2+^ are blue and green, respectively).

The Schiff-base ligand H_2_L was prepared according to literature method (Lam et al., 1996). In the presence of Et_3_N, reactions of H_2_L with Zn(OAc)_2_·2H_2_O and Ln(OAc)_3_·4H_2_O (Ln = Nd, Yb, and Sm) in refluxing methanol/ethanol produced yellow solutions and the yellow crystalline products of **1**-**3** were obtained by the diffusion of diethyl ether into the solutions. A Smart APEX CCD diffractometer is used to collect the crystal data of all clusters (Supporting information, X-Ray Crystallography) (Tables S1–S4 and Data sheet 2). **1**-**3** have similar square-like structures, and two views of the crystal structure of **1** are shown in Figure 1. The top view is looking right in front of the square while the lower one is essentially a side-on view. The molecular sizes of **1** are about 8 × 14 × 21 Å. The structure of **1** is centrally symmetric with two equivalent Nd_2_Zn_4_L(OAc)_10_(OH)_2_ moieties linked by two L ligands. In each Nd_2_Zn_4_L(OAc)_10_(OH)_2_ moiety, the Nd^3+^ ion is coordinated with nine oxygen atoms from five OAc^−^, two OH^−^ ions and one L ligand. Meanwhile, two Nd^3+^ ions are linked by two OH^−^ ions, and the Nd···Nd distance is 4.190 Å. All Zn^2+^ ions show similar tetrahedral geometries. For the OAc ^−^ anions, each one binds to one Nd^3+^ and one Zn^2+^ ion. Each OH^−^ anion bonds to one Zn^2+^ and two Nd^3+^ ions. In **1**, it is found that the L ligand is coordinated with two Nd^3+^ and two Zn^2+^ ions by its N and phenoxide and methoxy O atoms, while two backbone O atoms do not involve in the coordination. Each Nd^3+^ ion and its closest three Zn^2+^ ions are bridged together through the L ligand, OAc^−^ anions and/or OH^−^ anions. The Nd···Zn distances range from 3.650 to 3.812 Å.

**Figure 2:**
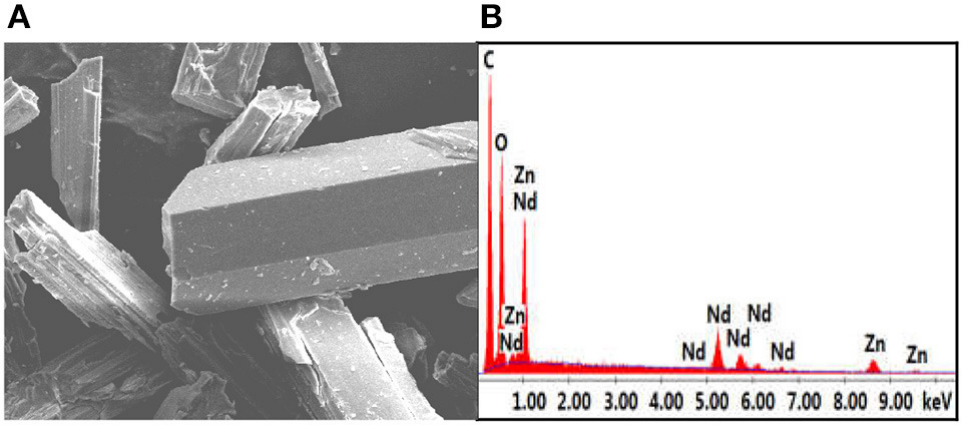
The SEM image **(A)** and EDX spectrum **(B)** of **1**.

In **1**, the bond lengths of Zn-O and Nd-O are 1.942–2.013 Å and 2.392–2.716 Å, respectively. The crystalline morphology of **1** was detected by a panoramic scanning electron microscopy (SEM) (Figure 2A). The Zn:Nd ratio in **1** is determined to be about 2:1 by energy dispersive X-ray spectroscopy (EDX) analysis, consistent with its crystal structure (Figure 2B).

**Figure 3:**
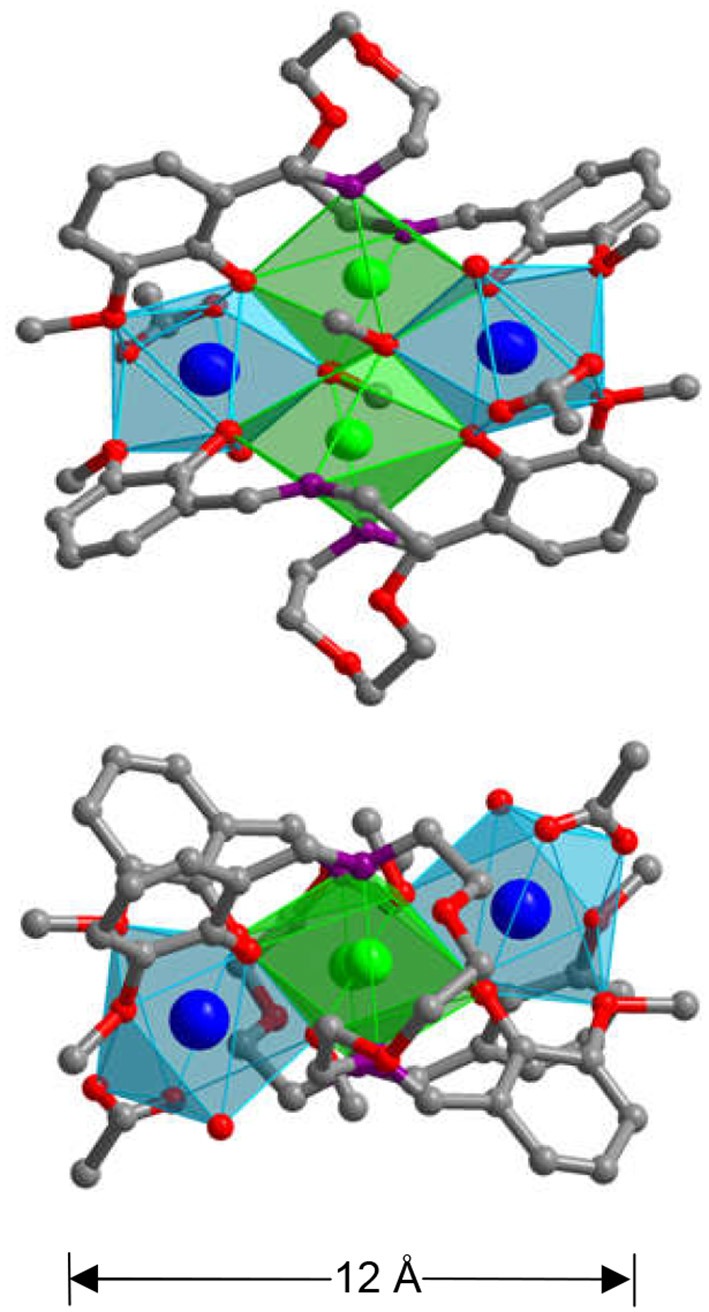
The square-like crystal structure of **4** (**Top**: viewed along the *a*-axis; **Lower**: viewed along the *c*-axis. The color of Nd^3+^ and Cd^2+^ are blue and green, respectively).

**Figure 4:**
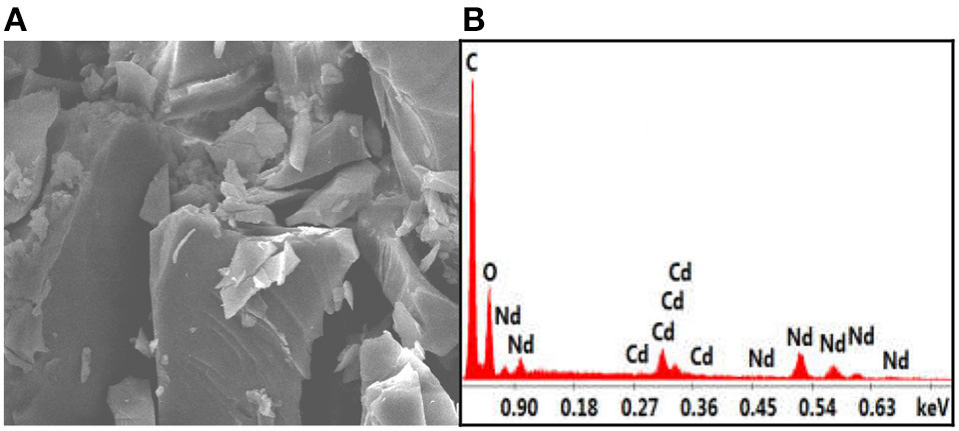
The SEM image **(A)** and EDX spectrum **(B)** of **4**.

Reactions of H_2_L with Cd(OAc)_2_·2H_2_O and Ln(OAc)_3_·4H_2_O (Ln = Nd, Yb, and Sm) in similar reaction conditions as above produced yellow crystalline products of **4**-**6** (Tables S5–S7 and Data sheet 2). The crystal structure of **4** is shown in Figure 3. The metric dimensions of **4** measure ~8 × 12 × 12 Å, which are smaller than those of **1**. As shown in Figure 3, the structure of **4** is also centrally symmetric with two equivalent NdCdL(OAc)(OH)(OCH_3_) moieties linked by two (OCH_3_)^−^ anions. In each NdCdL(OAc)(OH)(OCH_3_) moiety, the Nd^3+^ ion is coordinated with eight oxygen atoms from two L ligands, one OAc^−^, one OH^−^, and one (OCH_3_)^−^ anions. The Cd^2+^ ion has an octahedral geometry. The Nd^3+^ and Cd^2+^ ions are linked by one L ligand and one (OCH_3_)^−^ anion with a separation of 3.656 Å. Each (OCH_3_)^−^ anion bonds to one Cd^2+^ and two Nd^3+^ ions. As found in **1**, two backbone O atoms of the L ligand are also not coordinated with the metals in **4**. Two Cd^2+^ ions are linked by two (OCH_3_)^−^ anions, and the Cd···Cd distance is 3.573 Å. In **4**, the bond lengths of Cd-O and Nd-O are 2.249–2.419 Å and 2.255–2.480 Å, respectively. The Cd:Nd ratio in **4** is found to be about 1:1 by energy dispersive X-ray spectroscopy analysis, consistent with its crystal structure (Figures 4A,B).

Powder XRD studies of **1**-**6** show that their experimental patterns are similar to their simulated ones generated from single crystal X-ray data (Figure S3). On heating **1** and **6** before 100 ^o^C results in weight losses of 3–18% (thermogravimetric analysis, Figure S4), which is due to the escaption of the uncoordinated solvent molecules such as H_2_O, MeOH, and EtOH. The thermodynamically stabilities of the clusters were texted through melting point measurements. It is found that **1**-**6** start to discompose from 161 to 190^o^C (Figure S4). Molar conductivity studies show that **1**-**6** are neutral in solution, in agreement with their solid state structures.

### Photophysical properties

**Figure 5:**
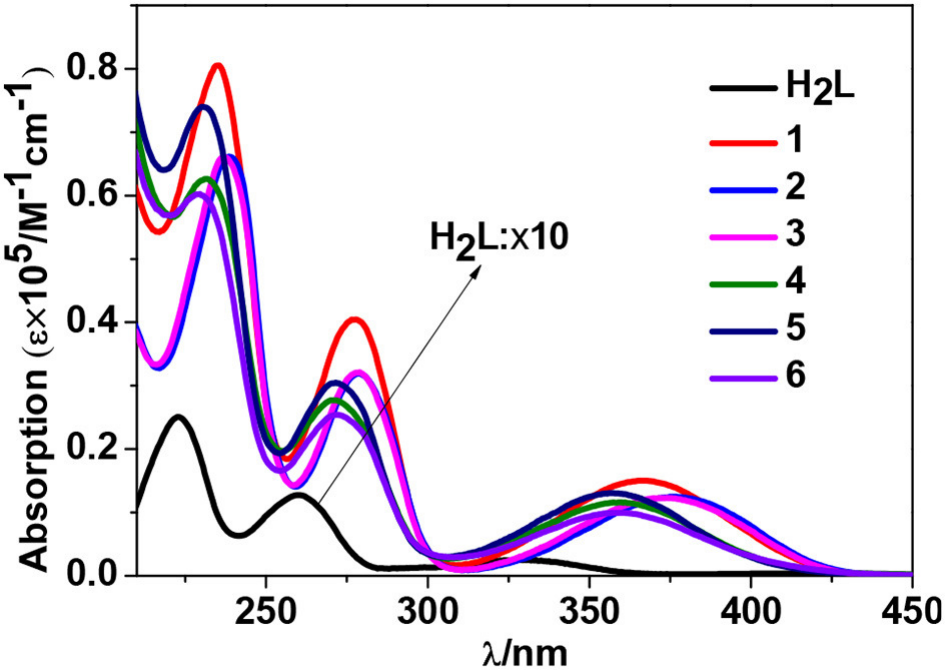
UV-Vis absorption spectra of the free Schiff base ligand H_2_L and **1**-**6** in CH_3_CN. (C = 10^−6^-10^−5^ M).

**Figure 6:**
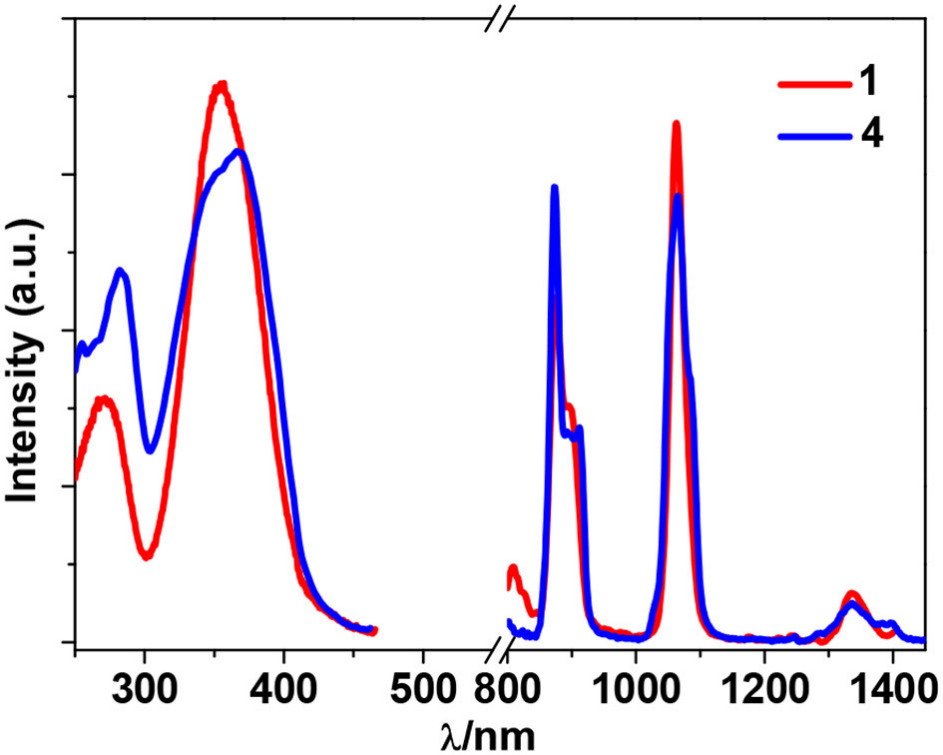
Excitation and emission spectra for **1** and **4** in CH_3_CN.

**Figure 7:**
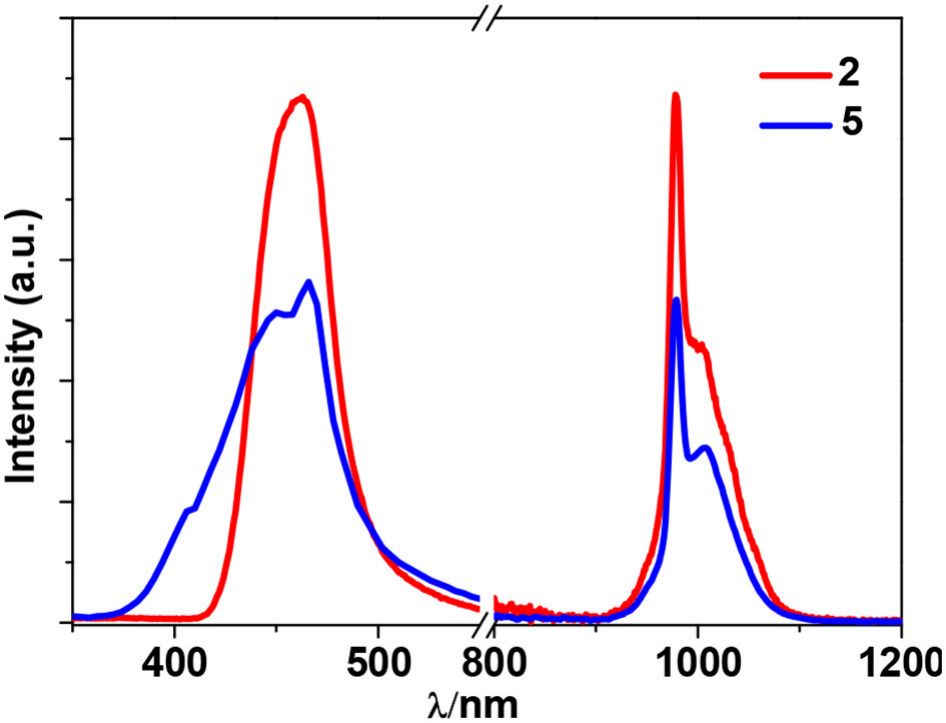
Excitation and emission spectra for **2** and **5** in CH_3_CN.

**Figure 8:**
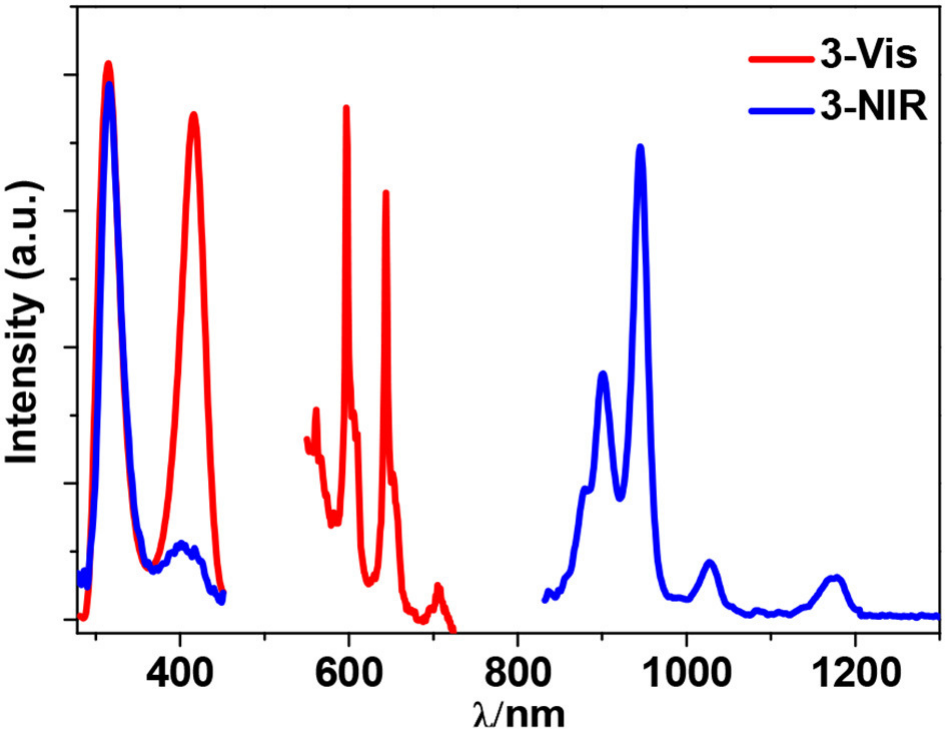
Excitation and emission spectra for **3** in CH_3_CN.

**Figure 9:**
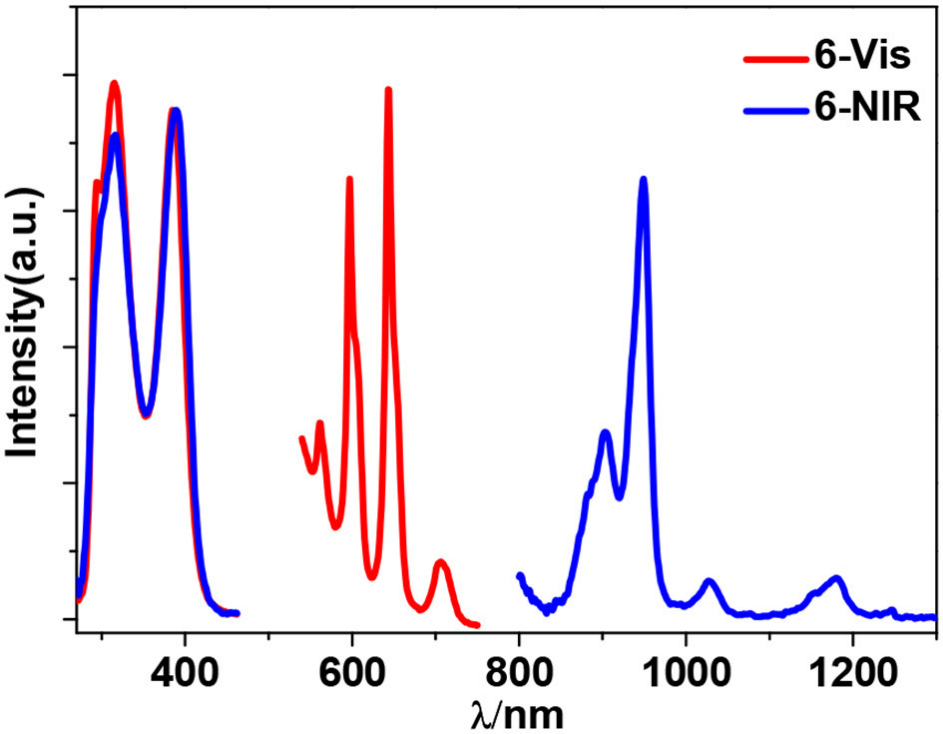
Excitation and emission spectra for **6** in CH_3_CN.

Zn^2+^ and Cd^2+^ ions have saturated d^10^ electronic configuration, which prevents the quenching of lanthanide luminescence through d-d transitions (i.e., f → d energy transfer) (Wen et al., 2007; Jankolovits et al., 2011). Thus, the light-absorbing Zn(II) and Cd(II) chromophores can be used as sensitizers for lanthanide emission. In order to obtain strongly luminescent lanthanide complexes, the chromophoric ligands which coordinate with the lanthanide metals should be able to absorb energy and transfer it efficiently to the central metals (“Antenna Effect”). For the efficiency of energy transfer from the ligand to the lanthanide ion (**LMET**), the energy gap between the excitation states of the former (donor) and latter (accepter) may play a key role (María et al., 2017). The photophysical properties of **1**-**6** were studied in CH_3_CN solution and the solid state. A FLS 980 fluorimeter was used to record luminescence spectra in the visible and NIR regions (Supporting information, Photophysical Studies). The UV-visible absorption spectrum of the free ligand H_2_L shows three bands at 222, 260, and 330 nm. These bands are found to be red-shifted in **1**-**6** (Figure 5). The free ligand exhibits emission bands at 416, 429, and 493 nm when excited with 280 or 378 nm light (Figure S5 in the ESI). Excited by ligand-centered absorption bands, **1** and **4** show typical NIR luminescence of Nd^3+^ (^4^${\text{F}}_{3/2}\to^{4}$I_j/2_ transitions, j = 9, 11, and 13), **2** and **5** show that of Yb^3+^ (^2^${\text{F}}_{5/2}\to^{2}$F_7/2_ transition), while **3** and **6** show visible and NIR emission spectra for Sm^3+^ (^4^${\text{G}}_{5/2}\to^{6}$H_j/2_ transitions, j = 5, 7, 9, and 11; ^4^${\text{G}}_{5/2}\to^{6}$F_j/2_ transitions, j = 1, 3, 5, 7, and 9) (Figures 6–**9**). Each d-f cluster shows similar luminescence spectra in the solution and the solid state. For **1**-**6**, the excitation wavelengths (λ_ex_) and molar absorption coefficients (ε), emission lifetimes (τ), quantum yields (Φ_em_) and the energy transfer efficiencies (η_sens_) are shown in Table 1.

**Table 1:** The excitation wavelengths (λ_ex_) and molar absorption coefficients (ε), emission lifetimes (τ), quantum yields (Φ_em_), and the energy transfer efficiencies (η_sens_) of **1**-**6** in CH_3_CN (^*a*^ Visible emission, ^*b*^ NIR emission).

**cluster**	**λ_ex_ (nm) / ε (M^−1^ cm^−1^)**	**τ (μs)**	**Φ_em_ (%)**	**η_sens_ (%)**
1	270/0.40, 355/0.15	5.06	0.43	21.28
2	462/–	8.50	0.33	76.74
3	316/0.01,*^*a*^*416/0.03*^*a*^*	25.37^*a*^	0.51^*a*^	60.0^*a*^
	315/0.01,*^*b*^* 401/0.07*^*b*^*	24.68^*b*^	0.03^*b*^	–
4	282/0.22, 367/0.11	6.33	0.35	13.83
5	466/–	11.73	0.39	66.10
6	315/0.03,*^*a*^* 387/0.06*^*a*^*	31.12^*a*^	0.41^*a*^	39.42^*a*^
	316/0.04,*^*b*^* 388/0.06*^*b*^*	32.01^*b*^	0.36^*b*^	–

**Scheme 2:**
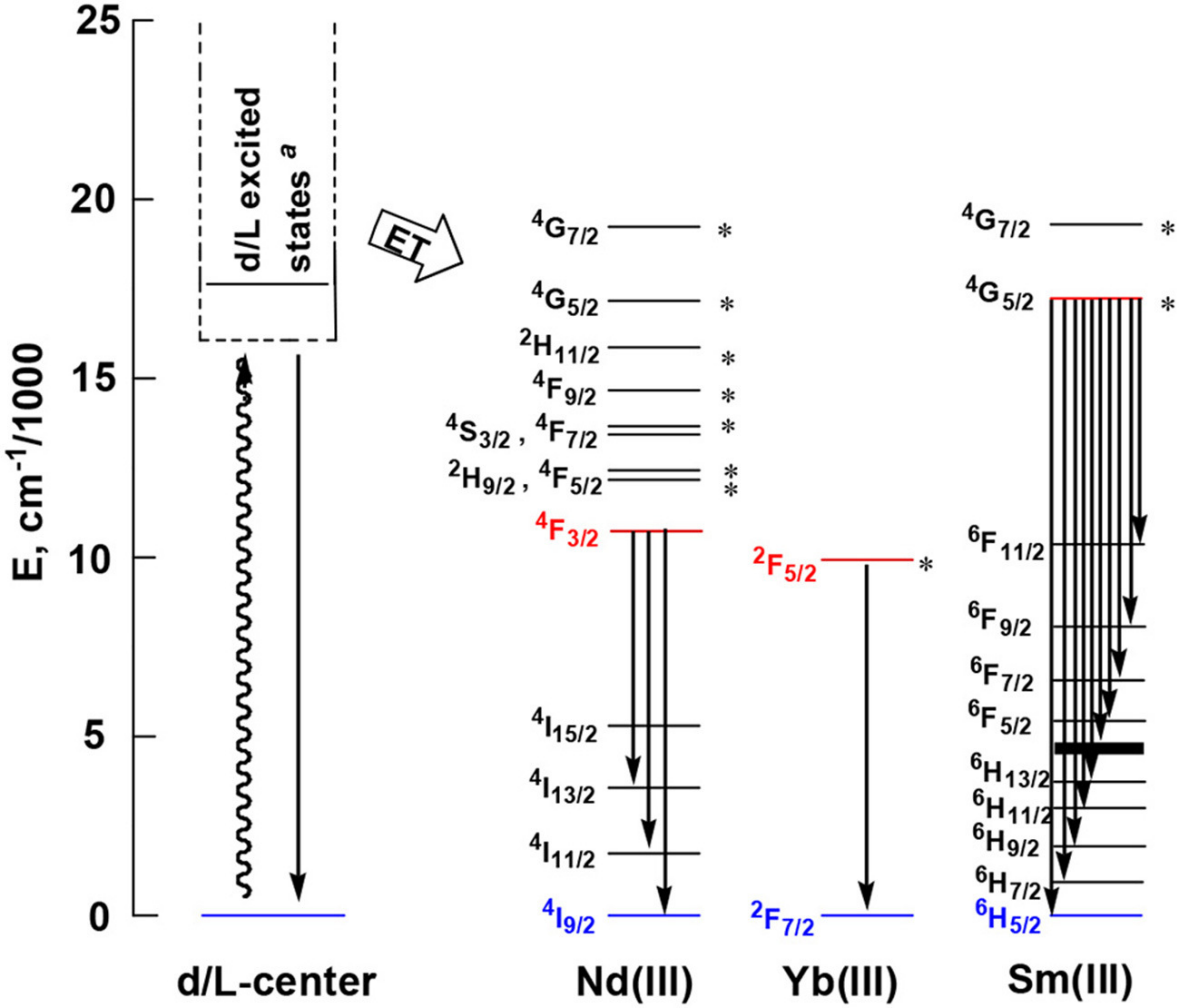
Energy level diagram of lanthanide ions in **1**-**6** (The excited states marked with ^*^ can accept energy from d/L center by either Förster or Dexter mechanism) (Bünzli and Piguet, 2005).

As shown in Figure 6, both **1** and **4** display NIR emission bands of Nd^3+^ at about 872, 1,065, and 1,334 nm. These two clusters have similar excitation spectra with two bands (λ_ex_ = 270–367 nm), in agreement with their absorption spectra, confirming that the energy transfers from the Zn/L or Cd/L centers to Nd^3+^ ions occur (**Scheme 2**). For either **1** or **4**, the intensity of excitation band at the long wavelength (i.e., 355 nm for **1** or 367 nm for **4**) is higher than that at the short wavelength (i.e., 270 nm for **1** or 282 nm for **4**). For Nd(III) complexes, the Nd^3+^ ion has many excitation energy levels lying above the emissive ^4^F_3/2_ state at 11,300 cm^−1^, which is helpful for the lanthanide ion to accept energy from the d/L center (**Scheme 2**) (Bünzli and Piguet, 2005; Shavaleev et al., 2005). The emission lifetimes (τ) of **1** and **4** are found to be 5.06 and 6.33 μs, respectively (Figure S6). The intrinsic quantum yields (Φ_Ln_) of Nd^3+^ emission in **1** and **4** are calculated as 2.02 and 2.53%, respectively, using Φ_Ln_ = τ/τ_0_ (τ_0_ = 250 μs (Meshkova et al., 1999), the natural lifetime of Nd^3+^). The emission quantum yields (Φ_em_) of **1** and **4** are measured as 0.43 and 0.35%, respectively. So the efficiencies (η_sens_) of the energy transfer from Zn/L- and Cd/L-center to Nd^3+^ in **1** and **4** are estimated to be 21.28 and 13.83%, respectively, using η_sens_ = Φ_em_/Φ_Ln_ (Bünzli and Piguet, 2005). This indicates that the Zn/L center in **1** has higher energy transfer efficiency than Cd/L center in **4**. The emission quantum yield of **1** is also bigger than **4** (0.43 vs. 0.35%). It is found that the absorption at the excitation wavelength in **1** (ε = 0.15 × 10^5^ M^−1^ cm^−1^ at 355 nm) is bigger than that in **4** (ε = 0.11 × 10^5^ M^−1^ cm^−1^ at 367 nm), which may be the cause of their differences in luminescence properties.

**2** and **5** exhibit NIR emission bands of Yb^3+^ at about 978 nm (Figure 7). They show one excitation band at 462 and 466 nm, respectively, where the clusters have no or very weak absorption (Figure 5). Differing from Nd^3+^ ion, Yb^3+^ ion has only a single excited state ^2^F_5/2_ at 10,200 cm^−1^ that is lower than those of Zn/L and Cd/L centers (**Scheme 2**) (Horrocks et al., 1997; Reinhard and Gudel, 2002). The emission lifetimes (τ) of **2** and **5** are found to be 8.50 and 11.73 μs, respectively (Figure S6). The intrinsic quantum yields (Φ_Ln_) of Yb^3+^ emission in **2** and **5** are calculated as 0.43 and 0.59%, respectively (Bünzli and Piguet, 2005) (the natural lifetime of Yb^3+^ is 2,000 μs). The emission quantum yields (Φ_em_) of **2** and **5** in CH_3_CN are measured as 0.33 and 0.39%, respectively. Thus, the efficiencies (η_sens_) of the energy transfer in **2** and **5** are estimated to be 76.74 and 66.10%, respectively (Bünzli and Piguet, 2005).

**Figure 10:**
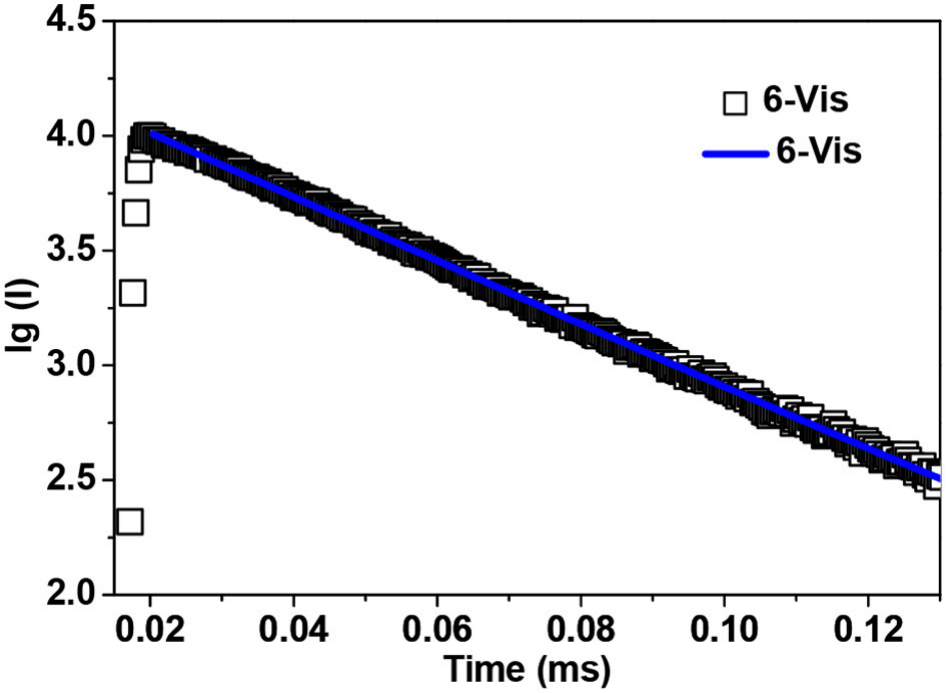
The visible emission lifetime of **6** in CH_3_CN.

Sm^3+^ ion may show emission bands both in the visible (^4^${\text{G}}_{5/2}\to^{6}$H_J_) and in the NIR (^4^${\text{G}}_{5/2}\to^{6}$F_J_) ranges (Scheme 2) (Chow et al., 2016). However, due to non-radiative loss attributed to multiphonon emission, Sm(III) complexes often display weak luminescence (Sabbatini et al., 2011). For **3** and **6** (Figures 8, 9), the hypersensitive transitions ^4^${\text{G}}_{5/2}\to^{6}$H_7/2_ and ^4^${\text{G}}_{5/2}\to^{6}$H_9/2_ are found at about 595 and 650 nm, respectively, which are responsible for the most intense lines in the visible region. A peak located at 561 nm (^4^${\text{G}}_{5/2}\to^{6}$H_5/2_ transition) has predominant magnetic dipolar character. The intensity ratio of *I*(^4^${\text{G}}_{5/2}\to^{6}$H_9/2_)/*I*(^4^${\text{G}}_{5/2}\to^{6}$H_5/2_) can be used as a measure for the polarizability of the chemical environment of the Sm^3+^ ion. For **3** and **6** they are calculated to be 6.28 and 5.20, respectively, which are comparable to those found in the literature (Lunstroot et al., 2009; Sun et al., 2013). The visible emission lifetimes (τ) of **3** and **6** are 25.37 and 31.12 μs in CH_3_CN, respectively (Figure 10, Figure S6), which are a little shorter than the value of [ZnSmL^b^(NO_3_)_3_(H_2_O)] complex reported by Andruh et al. (Pasatoiu et al., 2011), but longer than those reported for some other Sm(III)-based complexes (Chen et al., 2005; Fomina et al., 2014; Foucault-Collet et al., 2014). The intrinsic quantum yields (Φ_Ln_) of Sm^3+^ emissions in **3** and **6** are calculated as 0.85 and 1.04%, respectively (the natural lifetime of Sm^3+^ is 3.0 ms; Malba et al., 2015). The visible emission quantum yields (Φ_em_) of **3** and **6** are found to be 0.51 and 0.41%, respectively. So the efficiencies (η_sens_) of the energy transfer from Zn/L- and Cd/L-center to Sm^3+^ are estimated to be 60.0 and 39.42%, respectively (Bünzli and Piguet, 2005). This indicates that the Zn/L center in **4** exhibits higher energy transfer efficiency than the Cd/L center in **6**. For either **4** or **6**, the most intense line in the NIR area is found at about 960 nm (^4^${\text{G}}_{5/2}\to^{6}$F_5/2_ transition), with a long NIR emission lifetime (τ) up to 24.68 μ*s* for **4** or 32.01 μs for **6** recorded in CH_3_CN.

The emission of the Schiff base ligand is also found in the visible emission spectra of **3** and **6**, indicating the energy transfer from Zn/L- and Cd/L-center to Sm^3+^ is not complete and the emission of Sm^3+^ is not strong enough to conceal the emission of the ligand. As we know, the coordinated OH^−^ anions in the clusters are closed to the lanthanide ions and can partially quench their emission (Richardson, 1982; Yanagida et al., 1998). As shown in Figures 8, 9, for either visible or NIR emission, **3** and **6** exhibit two excitation bands (λ_ex_ = 315–416 nm, Table 1), which are from ligand-centered excited states. For **6**, these two excitation bands have similar intensities. It is noticeable that, for the NIR emission of **3**, the intensity of the excitation band at 315 nm is much higher than that at 401 nm, indicating that the NIR luminescence of **3** is dominated by the former. However, the absorption of **3** at 315 nm is very low (ε = 0.01 × 10^5^ M^−1^ cm^−1^), which is not advantageous for the ligand-center to absorb energy for sensitizing the lanthanide luminescence. As shown in Table 1, the NIR emission quantum yield of **3** is found to be only 0.03%, which is more than ten times less than that of **6**. Thus, the ability of these clusters to absorb energy at the excitation wavelengths can efficiently affect their luminescence properties. We were naturally interested in the difference in the luminescence properties between the Zn-Ln clusters formed by H_2_L and H_2_L^f^ (**Scheme 1B**). It is found that, the Zn-Nd cluster **1** has a lower energy transfer efficiency (η_sens_) than [Zn_8_Nd_4_(L^f^)_8_(OAc)_8_](OH)_4_ (21.28 vs. 39.89%), while the η_sens_ value of the Zn-Sm cluster **3** is similar as [Zn_8_Sm_4_(L^f^)_8_(OAc)_8_](OH)_4_ (60.0 vs. 58.24%) (Bo et al., 2018).

**Figure 11:**
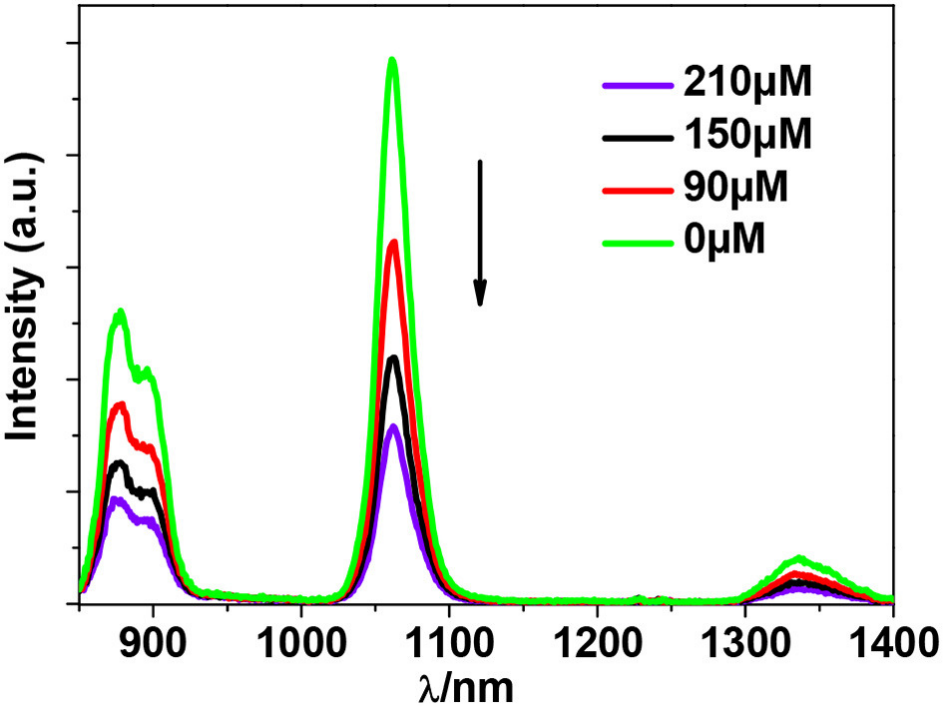
Decrease in the luminescence intensity of **1** in DMF upon the addition of different concentrations of Cu(OAc)_2_.

In additional, the d metal ions may perturb the electronic structure of the ligand and affect its singlet and triplet excited states. These changes, in turn, can also affect how effectively the emissive states of the Ln^3+^ ion are sensitized by the ligand (Tang et al., 2013), resulting in chelation enhancement of the quenching (CHEQ) or chelation enhancement of the fluorescence emission (CHEF). The influence of Cu^2+^ ion on the luminescence of **1** was investigated in DMF. The intensities of the NIR emission of **1** were recorded as the Cu^2+^ ion was added with different concentration. Interestingly, the addition of Cu^2+^ ion resulted in the decrease of the emission intensities (Figure 11). The Cu^2+^ ion has an unsaturated d electronic configuration (d^9^) and may quench the luminescence through d-d transitions (i.e., f → d energy transfer, CHEQ) (Wen et al., 2007; Jankolovits et al., 2011).

## Conclusions

In summary, six Zn-Ln and Cd-Ln (Ln = Nd, Yb, and Sm) square-like clusters were constructed successfully from a flexible long-chain Schiff base ligand featuring a long (CH_2_)_2_O(CH_2_)_2_O(CH_2_)_2_ chain backbone. These clusters are of nanoscale proportions (i.e., 8 × 14 × 21 Å for **1**), with the Schiff base ligands showing “linear” configurations. The study of luminescence properties shows that the Zn/L and Cd/L chromophores of the clusters can effectively transfer energy to the lanthanide ions, and the formers in **1**, **2**, and **3** have higher energy transfer efficiencies than the latters in **4**, **5**, and **6**, respectively. The luminescence properties of the clusters may be efficiently affected by some factors such as the presence of coordinated OH^−^ anions in the structures, the absorption at the excitation wavelengths, and the energy transfer efficiencies between the donors and accepters.

## Author contributions

XY and DS designed the experiments. TZ, HC, DJ and CW performed the experiments. SW, LB and XZ analyzed the data. TZ wrote the paper. XY, DS and XZ revised the paper.

### Conflict of interest statement

The authors declare that the research was conducted in the absence of any commercial or financial relationships that could be construed as a potential conflict of interest.
