# Supplementary material for: Luminescent Polynuclear Zn- and Cd-Ln Square-Like Nanoclusters With a Flexible Long-Chain Schiff Base Ligand
Source: Front Chem. 2018 Jul 31;6:321. doi: 10.3389/fchem.2018.00321 (PMC6080591; doi:10.3389/fchem.2018.00321)
Supplement: Supplementary file 1 [file Table_1.DOC]

**Table of Contents**

Luminescent Polynuclear Zn- and Cd-Ln Square-like Nanoclusters with a Flexible Long-chain Schiff Base Ligand

Ting Zhu, Xiaoping Yang, Shiqing Wang, Le Bo, Chengri Wang, Hongfen Chen, Dongmei Jiang and Desmond Schipper

Two series of Zn-Ln and Cd-Ln (Ln = Nd, Yb and Sm) rectangular nanoclusters were prepared using a long-chain Schiff base ligand, and their visible and NIR luminescence properties were determined.


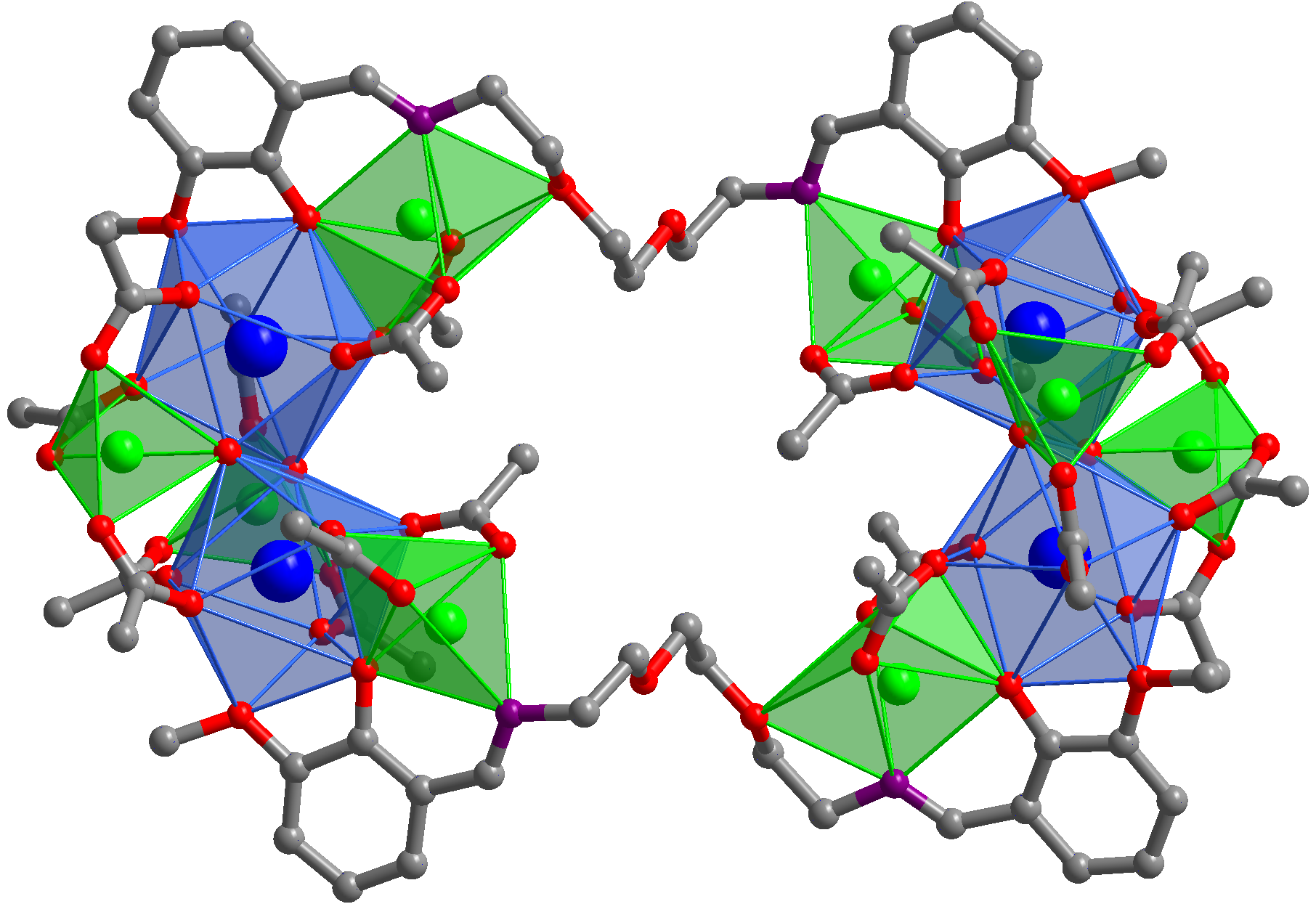


**Zn/Cd-Ln nanoclusters**

**Vis/NIR**

**UV-vis**
