# Supplementary material for: Luminescent Polynuclear Zn- and Cd-Ln Square-Like Nanoclusters With a Flexible Long-Chain Schiff Base Ligand
Source: Front Chem. 2018 Jul 31;6:321. doi: 10.3389/fchem.2018.00321 (PMC6080591; doi:10.3389/fchem.2018.00321)
Supplement: Supplementary file 2 [file Data_Sheet_1.DOC]

**Supporting Information**

Luminescent Polynuclear Zn- and Cd-Ln Square-like Nanoclusters with a Flexible Long-chain Schiff Base Ligand

Ting Zhu, Xiaoping Yang,* Shiqing Wang, Le Bo, Chengri Wang, Hongfen Chen, Dongmei Jiang and Desmond Schipper

**Contents**

1. Materials and General Methods.………….……………………………....…......…......….S1

2. The IR spectra of the free ligand H_2_L and clusters **1**-**6**..........................................….…….S2

3. The MS(ESI) spectra of **4** and **5**.………….……………………………....…......…......….S3

4. Powder XRD patterns of clusters...................................................................…….….…....S4

5. The thermogravimetric analysis of **1**-**6**..…….….…........................................................….S6

6. Photophysical Studies……………….……………………………....…....…....….........….S8

7. Photophysical properties of the free ligand H_2_L and clusters **1**-**6**.….…….….…................S9

8. X-Ray Crystallography……………….……………………………....…....…....….......….S11

**1. Materials and General Methods**

All reactions were performed under dry oxygen-free dinitrogen atmospheres using standard Schlenk techniques. Physical measurements: NMR: AVANCE III AV500. 500 spectrometer (^1^H, 500 MHz) at 298 K; HRMS(ESI) analysis: MicroOTOF-QII; IR: Nicolet IS10 spectrometer; Powder XRD: D8ADVANCE. Elemental analyses (C, H, N) of compounds were carried out on a EURO EA3000 elemental analysis after dried in an oven at 100^o^C for 2 hours. Melting points were obtained in sealed glass capillaries under dinitrogen and are uncorrected. The thermogravimetric analyses were carried out on a TA Instruments Q600. Absorption spectra were obtained on a UV-3600 spectrophotometer, and excitation and emission spectra on a FLS 980 fluorimeter.

**2. The IR spectra of the free ligand H_2_L and clusters 1-6**

**Figure S1**. The IR spectra of the free ligand H_2_L and clusters **1**-**6.**

**3. The MS(ESI) spectra of 4 and 5**

**Figure S2**. The MS(ESI) spectra of **4** and **5**.

**4. Powder XRD patterns of clusters**


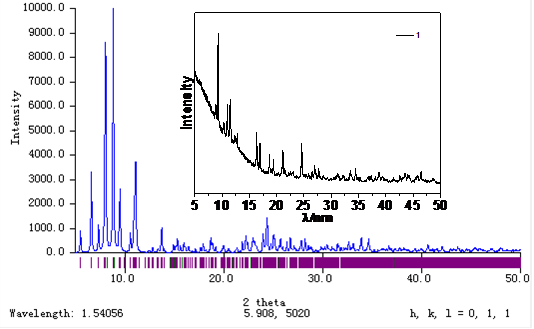


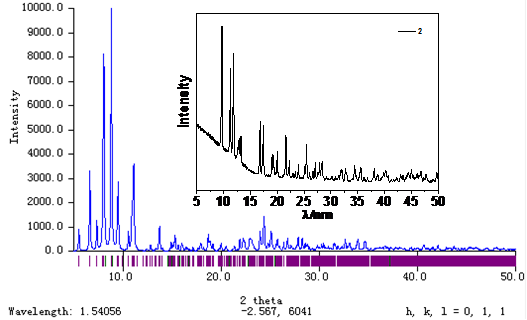


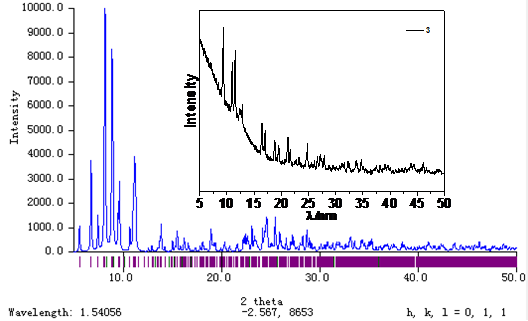


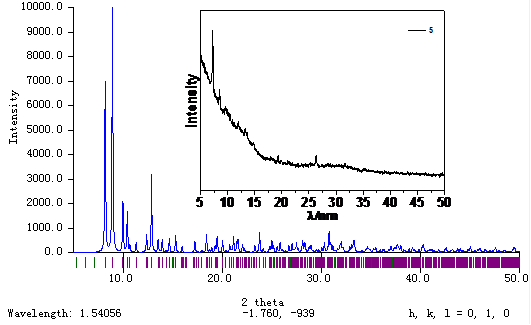


**Figure S3**. Powder XRD patterns of clusters (Insert: experimental patterns).

**5. The thermogravimetric analysis of 1-6**

**Figure S4**. The thermogravimetric analysis of **1**-**6**.

**6. Photophysical Studies**

The UV-visible absorption spectra were recorded at RT using an UV-3600 spectrophotometer. The solvent employed was of HPLC grade. The wavelength range was set from 600 to 200 nm. Luminescence spectra in the visible and NIR regions were recorded on a FLS 980 fluorimeter. The light source for excitation and emission spectra was a 450 W xenon arc lamp with continuous spectral distribution from 190 to 2600 nm. Liquid nitrogen cooled Ge PIN diode detector was used to detect the NIR emissions from 800 nm to 1700 nm. The temporal decay curves of the fluorescence signals were stored by using the attached storage digital oscilloscope. Photoluminescence lifetimes were determined by the TCSPC (time-correlated single-photon counting) method. The overall quantum yields (Φ_em_) were obtained by using an integrating sphere, according to eqn Φ_em_ = *N*_em_ / *N*_abs_, where *N*_em_ and *N*_abs_ are the numbers of emitted and absorbed photons, respectively. The intrinsic quantum yields (Φ_Ln_) of Ln^3+^ emission are calculated using Φ_Ln_ = *τ*/*τ*_0_, where *τ* and *τ*_0_ are the observed emission lifetime and the natural lifetime of Ln^3+^, respectively. Besides, systematic errors have been deducted through the standard instrument corrections. All the measurements were carried out at room temperature.

**7. Photophysical properties of the free ligand H_2_L and clusters 1-6**


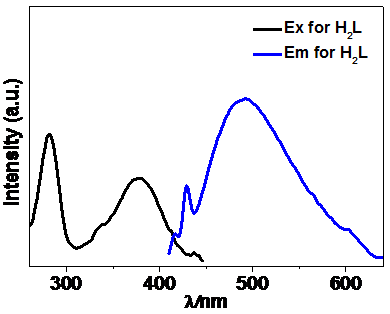


**Figure S5.** Excitation and emission spectra of free ligands H_2_L in CH_3_CN.

**Figure S6.** The lifetimes of clusters **1**-**6** in CH_3_CN.

8. X-Ray Crystallography

Data were collected on a Smart APEX CCD diffractometer with graphite monochromated Mo-K*α* radiation (*λ* = 0.71073 Å) at 190 K. The data set was corrected for absorption based on multiple scans and reduced using standard methods. Data reduction was performed using DENZO-SMN. The structures were solved by direct methods and refined anisotropically using full-matrix least-squares methods with the SHELX 97 program package. Coordinates of the non-hydrogen atoms were refined anisotropically, while hydrogen atoms were included in the calculation isotropically but not refined. Neutral atom scattering factors were taken from Cromer and Waber. Crystallographic data for **1**-**6** have been deposited with the Cambridge Crystallographic Data (CCDC reference numbers 1568089-1568094). These data can be obtained free of charge via www.ccdc.cam.ac.uk/data_request/cif. Crystallographic data for **1**-**6** are presented in Table S1 and selected bond lengths and angles are given in Tables S2-S7.

| **Clusters** | **1** | **2** | **3** | **4** | **5** | **6** |
| --- | --- | --- | --- | --- | --- | --- |
| Formula | C_88_H_128_N_4_  O_60_Zn_8_Nd_4_ | C_88_H_128_N_4_  O_60_Zn_8_Yb_4_ | C_88_H_128_N_4_  O_60_Zn_8_Sm_4_ | C_50_H_66_N_4_  O_20_Cd_2_Nd_2_ | C_50_H_66_N_4_  O_20_Cd_2_Yb_2_ | C_50_H_66_N_4_  O_20_Cd_2_Sm_2_ |
| Fw | 3301.86 | 3417.06 | 3326.30 | 1556.35 | 1613.95 | 1568.57 |
| Crystal system | Monoclinic | Monoclinic | Monoclinic | Monoclinic | Monoclinic | Monoclinic |
| Space group | P2(1)/c | P2(1)/c | P2(1)/c | P2(1)/n | P2(1)/n | P2(1)/n |
| *a* [Ǻ] | 12.077(4) | 12.0804(4) | 11.929(2) | 12.377(4) | 12.4151(6) | 12.387(2) |
| *b* [Ǻ] | 26.643(9) | 26.5935(8) | 26.173(5) | 16.508(5) | 16.5360(8) | 16.580(3) |
| *c* [Ǻ] | 20.099(7) | 20.1084(5) | 20.016(3) | 14.290(5) | 14.3093(7) | 14.324(2) |
| *α* [deg] | 90 | 90 | 90 | 90 | 90 | 90 |
| *β* [deg] | 95.486(6) | 95.477(2) | 95.430(4) | 95.849(6) | 96.345(10) | 95.789(4) |
| *γ* [deg] | 90 | 90 | 90 | 90 | 90 | 90 |
| *V* / [Ǻ^3^] | 6438(4) | 6430.05(3) | 6221.3(19) | 2904.6(17) | 2919.6(2) | 2926.9(8) |
| d / [g/cm^3^] | 1.703 | 1.765 | 1.776 | 1.780 | 1.836 | 1.780 |
| *Z* | 2 | 2 | 2 | 2 | 2 | 2 |
| *T* [K] | 190 | 190 | 190 | 190 | 190 | 190 |
| F(000) | 3288 | 3368 | 3304 | 1540 | 1580 | 1548 |
| *μ*, mm^-1^ | 3.132 | 4.429 | 3.459 | 2.555 | 3.967 | 2.768 |
| *θ* rang, deg | 1.69-25.00 | 2.04-25.00 | 1.28-25.00 | 1.89-25.00 | 1.89-25.00 | 1.88-25.00 |
| reflns meads | 47800 | 18586 | 46025 | 8773 | 21589 | 21099 |
| reflns used | 11280 | 11018 | 10913 | 4885 | 5119 | 5133 |
| params | 739 | 739 | 739 | 352 | 352 | 352 |
| R_1_*^a^* [*I* >2*σ*(*I*)] | 0.0417 | 0.1176 | 0.0567 | 0.0819 | 0.0639 | 0.0594 |
| wR_2_*^a^* [*I* >2*σ*(*I*)] | 0.1157 | 0.2714 | 0.1400 | 0.2507 | 0.2319 | 0.2119 |
| Quality of fit | 1.069 | 2.094 | 1.036 | 1.335 | 1.091 | 1.059 |
| *^a^* R_1_ = Σ|*F*_o_| – |*F*_c_|Σ|*F*_o_|. wR_2_ = [Σ*w*[(*F*_o_^2^ – *F*_c_^2^)^2^]/Σ|[*w*(*F*_o_^2^)^2^]]^1/2^. *w*=1/[*σ*^2^(*F*_o_^2^)+(0.075*P*)^2^],  where *P* = [max(*F*_o_^2^,0)+2*F*_c_^2^]/3. | | | | | | |

**Table S1.** Crystal data and structure refinement for **1**-**6**.

**Table S2**. Selected Bond Lengths (Å) and Angles (°) for **1**.

Nd(1)-O(15) 2.399(6)

Nd(1)-O(11) 2.440(5)

Nd(1)-O(25) 2.466(5)

Nd(1)-O(13) 2.472(5)

Nd(1)-O(10) 2.472(5)

Nd(1)-O(2) 2.494(5)

Nd(1)-O(26) 2.509(5)

Nd(1)-O(27) 2.515(6)

Nd(1)-O(1) 2.715(5)

Nd(2)-O(18) 2.392(6)

Nd(2)-O(26) 2.466(5)

Nd(2)-O(24) 2.472(6)

Nd(2)-O(28) 2.479(5)

Nd(2)-O(22) 2.480(5)

Nd(2)-O(5) 2.497(5)

Nd(2)-O(20) 2.500(6)

Nd(2)-O(25) 2.528(4)

Nd(2)-O(6) 2.716(6)

Zn(1)-O(7) 1.946(6)

Zn(1)-O(2) 1.965(5)

Zn(1)-O(9) 1.976(6)

Zn(1)-N(1) 2.003(7)

Zn(2)-O(19) 1.942(7)

Zn(2)-O(26) 1.943(5)

Zn(2)-O(12) 1.952(7)

Zn(2)-O(16) 1.961(7)

Zn(3)-O(25) 1.947(5)

Zn(3)-O(14) 1.952(6)

Zn(3)-O(17) 1.969(7)

Zn(3)-O(23) 1.970(6)

Zn(4)-O(21) 1.957(6)

Zn(4)-O(8) 1.960(6)

Zn(4)-N(2) 2.005(7)

Zn(4)-O(5) 2.013(5)

O(15)-Nd(1)-O(11) 78.3(2)

O(15)-Nd(1)-O(25) 81.51(18)

O(11)-Nd(1)-O(25) 138.18(17)

O(15)-Nd(1)-O(13) 76.4(2)

O(11)-Nd(1)-O(13) 133.13(19)

O(25)-Nd(1)-O(13) 75.09(16)

O(15)-Nd(1)-O(10) 141.54(19)

O(11)-Nd(1)-O(10) 139.9(2)

O(25)-Nd(1)-O(10) 69.92(17)

O(13)-Nd(1)-O(10) 71.9(2)

O(15)-Nd(1)-O(2) 131.14(18)

O(11)-Nd(1)-O(2) 78.87(19)

O(25)-Nd(1)-O(2) 139.58(16)

O(13)-Nd(1)-O(2) 89.09(18)

O(10)-Nd(1)-O(2) 69.83(17)

O(15)-Nd(1)-O(26) 76.58(18)

O(11)-Nd(1)-O(26) 74.63(17)

O(25)-Nd(1)-O(26) 65.19(15)

O(13)-Nd(1)-O(26) 134.44(17)

O(10)-Nd(1)-O(26) 111.72(17)

O(2)-Nd(1)-O(26) 135.89(16)

O(15)-Nd(1)-O(27) 137.56(19)

O(11)-Nd(1)-O(27) 74.4(2)

O(25)-Nd(1)-O(27) 97.88(17)

O(13)-Nd(1)-O(27) 144.9(2)

O(10)-Nd(1)-O(27) 73.4(2)

O(2)-Nd(1)-O(27) 74.04(17)

O(26)-Nd(1)-O(27) 65.26(18)

O(15)-Nd(1)-O(1) 71.84(18)

O(11)-Nd(1)-O(1) 68.02(17)

O(25)-Nd(1)-O(1) 137.44(15)

O(13)-Nd(1)-O(1) 66.87(17)

O(10)-Nd(1)-O(1) 113.16(17)

O(2)-Nd(1)-O(1) 59.63(16)

O(26)-Nd(1)-O(1) 134.81(16)

O(27)-Nd(1)-O(1) 124.21(18)

O(18)-Nd(2)-O(26) 84.41(19)

O(18)-Nd(2)-O(24) 79.2(2)

O(26)-Nd(2)-O(24) 137.11(16)

O(18)-Nd(2)-O(28) 137.59(19)

O(26)-Nd(2)-O(28) 90.42(17)

O(24)-Nd(2)-O(28) 76.40(19)

O(18)-Nd(2)-O(22) 140.1(2)

O(26)-Nd(2)-O(22) 69.78(17)

O(24)-Nd(2)-O(22) 139.97(19)

O(28)-Nd(2)-O(22) 74.33(19)

O(18)-Nd(2)-O(5) 130.49(19)

O(26)-Nd(2)-O(5) 140.62(16)

O(24)-Nd(2)-O(5) 75.51(17)

O(28)-Nd(2)-O(5) 75.18(17)

O(22)-Nd(2)-O(5) 71.05(17)

O(18)-Nd(2)-O(20) 73.0(2)

O(26)-Nd(2)-O(20) 77.3(2)

O(24)-Nd(2)-O(20) 132.8(2)

O(28)-Nd(2)-O(20) 146.3(2)

O(22)-Nd(2)-O(20) 72.0(2)

O(5)-Nd(2)-O(20) 94.5(2)

O(18)-Nd(2)-O(25) 73.49(18)

O(26)-Nd(2)-O(25) 64.90(14)

O(24)-Nd(2)-O(25) 72.47(16)

O(28)-Nd(2)-O(25) 66.33(16)

O(22)-Nd(2)-O(25) 118.07(17)

O(5)-Nd(2)-O(25) 134.36(16)

O(20)-Nd(2)-O(25) 131.1(2)

O(18)-Nd(2)-O(6) 72.2(2)

O(26)-Nd(2)-O(6) 142.78(18)

O(24)-Nd(2)-O(6) 67.03(19)

O(28)-Nd(2)-O(6) 126.36(18)

O(22)-Nd(2)-O(6) 111.13(19)

O(5)-Nd(2)-O(6) 58.91(17)

O(20)-Nd(2)-O(6) 68.5(2)

O(25)-Nd(2)-O(6) 130.56(17)

O(7)-Zn(1)-O(2) 117.7(3)

O(7)-Zn(1)-O(9) 113.3(3)

O(2)-Zn(1)-O(9) 105.5(2)

O(7)-Zn(1)-N(1) 107.3(3)

O(2)-Zn(1)-N(1) 96.0(2)

O(9)-Zn(1)-N(1) 116.2(2)

O(19)-Zn(2)-O(26) 114.2(3)

O(19)-Zn(2)-O(12) 106.1(3)

O(26)-Zn(2)-O(12) 103.3(3)

O(19)-Zn(2)-O(16) 106.1(3)

O(26)-Zn(2)-O(16) 115.0(2)

O(12)-Zn(2)-O(16) 111.9(3)

O(25)-Zn(3)-O(14) 109.2(2)

O(25)-Zn(3)-O(17) 119.6(2)

O(14)-Zn(3)-O(17) 110.1(3)

O(25)-Zn(3)-O(23) 109.5(2)

O(14)-Zn(3)-O(23) 106.3(3)

O(17)-Zn(3)-O(23) 101.2(3)

O(21)-Zn(4)-O(8) 116.8(3)

O(21)-Zn(4)-N(2) 125.6(3)

O(8)-Zn(4)-N(2) 104.6(3)

O(21)-Zn(4)-O(5) 102.5(2)

O(8)-Zn(4)-O(5) 110.0(2)

N(2)-Zn(4)-O(5) 94.2(2)

**Table S3**. Selected Bond Lengths (Å) and Angles (°) for **2**.

Yb(1)-O(15) 2.381(19)

Yb(1)-O(13) 2.451(13)

Yb(1)-O(11) 2.452(15)

Yb(1)-O(10) 2.481(15)

Yb(1)-O(26) 2.498(12)

Yb(1)-O(2) 2.502(13)

Yb(1)-O(25) 2.504(13)

Yb(1)-O(27) 2.540(15)

Yb(1)-O(1) 2.736(16)

Yb(2)-O(18) 2.410(18)

Yb(2)-O(24) 2.427(16)

Yb(2)-O(22) 2.482(16)

Yb(2)-O(5) 2.493(15)

Yb(2)-O(28) 2.499(18)

Yb(2)-O(26) 2.514(13)

Yb(2)-O(20) 2.518(16)

Yb(2)-O(25) 2.533(12)

Yb(2)-O(6) 2.771(16)

Zn(1)-O(7) 1.930(16)

Zn(1)-O(2) 1.938(14)

Zn(1)-O(9) 1.993(14)

Zn(1)-N(1) 1.999(19)

Zn(2)-O(16) 1.903(17)

Zn(2)-O(26) 1.913(14)

Zn(2)-O(19) 1.932(16)

Zn(2)-O(12) 1.939(17)

Zn(3)-O(25) 1.926(13)

Zn(3)-O(14) 1.946(14)

Zn(3)-O(17) 1.955(17)

Zn(3)-O(23) 1.962(17)

Zn(4)-O(21) 1.943(13)

Zn(4)-O(8) 1.987(15)

Zn(4)-N(2) 2.011(19)

Zn(4)-O(5) 2.032(15)

O(15)-Yb(1)-O(13) 75.8(5)

O(15)-Yb(1)-O(11) 79.3(5)

O(13)-Yb(1)-O(11) 133.0(5)

O(15)-Yb(1)-O(10) 140.8(5)

O(13)-Yb(1)-O(10) 71.6(5)

O(11)-Yb(1)-O(10) 139.6(5)

O(15)-Yb(1)-O(26) 77.2(5)

O(13)-Yb(1)-O(26) 135.5(5)

O(11)-Yb(1)-O(26) 74.0(5)

O(10)-Yb(1)-O(26) 112.6(5)

O(15)-Yb(1)-O(2) 131.2(5)

O(13)-Yb(1)-O(2) 88.4(4)

O(11)-Yb(1)-O(2) 79.0(5)

O(10)-Yb(1)-O(2) 69.1(4)

O(26)-Yb(1)-O(2) 135.6(5)

O(15)-Yb(1)-O(25) 81.7(5)

O(13)-Yb(1)-O(25) 75.6(4)

O(11)-Yb(1)-O(25) 138.5(4)

O(10)-Yb(1)-O(25) 69.8(4)

O(26)-Yb(1)-O(25) 66.0(4)

O(2)-Yb(1)-O(25) 138.8(4)

O(15)-Yb(1)-O(27) 138.2(5)

O(13)-Yb(1)-O(27) 145.0(5)

O(11)-Yb(1)-O(27) 73.8(5)

O(10)-Yb(1)-O(27) 74.0(5)

O(26)-Yb(1)-O(27) 65.0(5)

O(2)-Yb(1)-O(27) 73.9(4)

O(25)-Yb(1)-O(27) 97.9(4)

O(15)-Yb(1)-O(1) 71.4(5)

O(13)-Yb(1)-O(1) 67.0(5)

O(11)-Yb(1)-O(1) 67.6(5)

O(10)-Yb(1)-O(1) 113.2(5)

O(26)-Yb(1)-O(1) 133.8(4)

O(2)-Yb(1)-O(1) 60.0(5)

O(25)-Yb(1)-O(1) 138.0(4)

O(27)-Yb(1)-O(1) 123.6(5)

O(18)-Yb(2)-O(24) 80.8(5)

O(18)-Yb(2)-O(22) 139.2(5)

O(24)-Yb(2)-O(22) 139.4(5)

O(18)-Yb(2)-O(5) 131.4(5)

O(24)-Yb(2)-O(5) 74.5(5)

O(22)-Yb(2)-O(5) 71.3(5)

O(18)-Yb(2)-O(28) 137.0(5)

O(24)-Yb(2)-O(28) 75.5(5)

O(22)-Yb(2)-O(28) 75.1(5)

O(5)-Yb(2)-O(28) 75.2(5)

O(18)-Yb(2)-O(26) 83.0(5)

O(24)-Yb(2)-O(26) 137.3(4)

O(22)-Yb(2)-O(26) 70.2(5)

O(5)-Yb(2)-O(26) 141.2(5)

O(28)-Yb(2)-O(26) 90.8(5)

O(18)-Yb(2)-O(20) 72.8(6)

O(24)-Yb(2)-O(20) 133.5(5)

O(22)-Yb(2)-O(20) 71.5(5)

O(5)-Yb(2)-O(20) 95.1(5)

O(28)-Yb(2)-O(20) 146.6(6)

O(26)-Yb(2)-O(20) 76.8(5)

O(18)-Yb(2)-O(25) 72.8(5)

O(24)-Yb(2)-O(25) 72.2(4)

O(22)-Yb(2)-O(25) 118.9(5)

O(5)-Yb(2)-O(25) 133.8(4)

O(28)-Yb(2)-O(25) 66.0(5)

O(26)-Yb(2)-O(25) 65.3(4)

O(20)-Yb(2)-O(25) 131.1(5)

O(18)-Yb(2)-O(6) 73.9(5)

O(24)-Yb(2)-O(6) 67.0(5)

O(22)-Yb(2)-O(6) 110.4(5)

O(5)-Yb(2)-O(6) 58.1(5)

O(28)-Yb(2)-O(6) 125.6(5)

O(26)-Yb(2)-O(6) 143.1(5)

O(20)-Yb(2)-O(6) 69.2(5)

O(25)-Yb(2)-O(6) 130.4(4)

O(7)-Zn(1)-O(2) 117.9(7)

O(7)-Zn(1)-O(9) 113.5(7)

O(2)-Zn(1)-O(9) 105.1(6)

O(7)-Zn(1)-N(1) 107.1(7)

O(2)-Zn(1)-N(1) 96.2(7)

O(9)-Zn(1)-N(1) 116.2(7)

O(16)-Zn(2)-O(26) 115.6(7)

O(16)-Zn(2)-O(19) 107.3(7)

O(26)-Zn(2)-O(19) 114.8(6)

O(16)-Zn(2)-O(12) 109.6(7)

O(26)-Zn(2)-O(12) 104.2(7)

O(19)-Zn(2)-O(12) 104.7(7)

O(25)-Zn(3)-O(14) 109.7(6)

O(25)-Zn(3)-O(17) 120.8(7)

O(14)-Zn(3)-O(17) 109.3(7)

O(25)-Zn(3)-O(23) 109.9(7)

O(14)-Zn(3)-O(23) 105.9(6)

O(17)-Zn(3)-O(23) 100.0(7)

O(21)-Zn(4)-O(8) 116.5(6)

O(21)-Zn(4)-N(2) 124.5(7)

O(8)-Zn(4)-N(2) 106.6(7)

O(21)-Zn(4)-O(5) 103.0(6)

O(8)-Zn(4)-O(5) 108.8(6)

N(2)-Zn(4)-O(5) 93.8(7)

**Table S4**. Selected Bond Lengths (Å) and Angles (°) for **3**.

Sm(1)-O(15) 2.412(8)

Sm(1)-O(11) 2.448(7)

Sm(1)-O(25) 2.476(7)

Sm(1)-O(2) 2.477(7)

Sm(1)-O(10) 2.481(7)

Sm(1)-O(13) 2.497(7)

Sm(1)-O(26) 2.512(7)

Sm(1)-O(27) 2.536(7)

Sm(1)-O(1) 2.675(7)

Sm(2)-O(18) 2.431(8)

Sm(2)-O(26) 2.479(7)

Sm(2)-O(22) 2.482(7)

Sm(2)-O(28) 2.486(7)

Sm(2)-O(24) 2.488(7)

Sm(2)-O(5) 2.490(7)

Sm(2)-O(20) 2.507(8)

Sm(2)-O(25) 2.519(6)

Sm(2)-O(6) 2.699(8)

Zn(1)-O(7) 1.960(8)

Zn(1)-O(2) 1.986(7)

Zn(1)-O(9) 1.993(7)

Zn(1)-N(1) 1.999(9)

Zn(2)-O(19) 1.952(8)

Zn(2)-O(26) 1.951(7)

Zn(2)-O(16) 1.971(8)

Zn(2)-O(12) 1.974(8)

Zn(3)-O(25) 1.944(7)

Zn(3)-O(14) 1.956(7)

Zn(3)-O(17) 1.971(8)

Zn(3)-O(23) 1.984(8)

Zn(4)-O(21) 1.962(7)

Zn(4)-O(8) 1.968(7)

Zn(4)-N(2) 2.001(9)

Zn(4)-O(5) 2.016(8)

O(15)-Sm(1)-O(11) 79.5(3)

O(15)-Sm(1)-O(25) 79.1(2)

O(11)-Sm(1)-O(25) 137.8(2)

O(15)-Sm(1)-O(2) 131.7(2)

O(11)-Sm(1)-O(2) 79.7(2)

O(25)-Sm(1)-O(2) 139.9(2)

O(15)-Sm(1)-O(10) 140.4(2)

O(11)-Sm(1)-O(10) 140.1(3)

O(25)-Sm(1)-O(10) 70.0(2)

O(2)-Sm(1)-O(10) 70.4(2)

O(15)-Sm(1)-O(13) 77.0(2)

O(11)-Sm(1)-O(13) 133.0(3)

O(25)-Sm(1)-O(13) 75.6(2)

O(2)-Sm(1)-O(13) 86.4(2)

O(10)-Sm(1)-O(13) 71.9(2)

O(15)-Sm(1)-O(26) 76.7(3)

O(11)-Sm(1)-O(26) 74.7(2)

O(25)-Sm(1)-O(26) 65.2(2)

O(2)-Sm(1)-O(26) 136.9(2)

O(10)-Sm(1)-O(26) 110.2(2)

O(13)-Sm(1)-O(26) 135.9(2)

O(15)-Sm(1)-O(27) 137.6(2)

O(11)-Sm(1)-O(27) 73.5(3)

O(25)-Sm(1)-O(27) 99.7(2)

O(2)-Sm(1)-O(27) 74.7(2)

O(10)-Sm(1)-O(27) 73.5(2)

O(13)-Sm(1)-O(27) 144.5(2)

O(26)-Sm(1)-O(27) 65.1(2)

O(15)-Sm(1)-O(1) 71.9(2)

O(11)-Sm(1)-O(1) 68.2(2)

O(25)-Sm(1)-O(1) 135.9(2)

O(2)-Sm(1)-O(1) 59.9(2)

O(10)-Sm(1)-O(1) 114.9(2)

O(13)-Sm(1)-O(1) 66.0(2)

O(26)-Sm(1)-O(1) 134.7(2)

O(27)-Sm(1)-O(1) 124.1(2)

O(18)-Sm(2)-O(26) 85.6(3)

O(18)-Sm(2)-O(22) 141.9(3)

O(26)-Sm(2)-O(22) 69.2(2)

O(18)-Sm(2)-O(28) 135.4(3)

O(26)-Sm(2)-O(28) 90.0(2)

O(22)-Sm(2)-O(28) 74.7(2)

O(18)-Sm(2)-O(24) 78.3(3)

O(26)-Sm(2)-O(24) 137.3(2)

O(22)-Sm(2)-O(24) 139.3(3)

O(28)-Sm(2)-O(24) 75.4(3)

O(18)-Sm(2)-O(5) 130.8(2)

O(26)-Sm(2)-O(5) 139.7(2)

O(22)-Sm(2)-O(5) 70.7(2)

O(28)-Sm(2)-O(5) 75.5(2)

O(24)-Sm(2)-O(5) 75.7(2)

O(18)-Sm(2)-O(20) 73.5(3)

O(26)-Sm(2)-O(20) 76.9(2)

O(22)-Sm(2)-O(20) 73.2(3)

O(28)-Sm(2)-O(20) 147.9(3)

O(24)-Sm(2)-O(20) 133.1(3)

O(5)-Sm(2)-O(20) 95.6(3)

O(18)-Sm(2)-O(25) 72.2(2)

O(26)-Sm(2)-O(25) 65.0(2)

O(22)-Sm(2)-O(25) 117.9(2)

O(28)-Sm(2)-O(25) 65.8(2)

O(24)-Sm(2)-O(25) 72.4(2)

O(5)-Sm(2)-O(25) 134.6(2)

O(20)-Sm(2)-O(25) 129.8(3)

O(18)-Sm(2)-O(6) 72.5(2)

O(26)-Sm(2)-O(6) 143.2(2)

O(22)-Sm(2)-O(6) 111.3(2)

O(28)-Sm(2)-O(6) 126.4(2)

O(24)-Sm(2)-O(6) 67.4(2)

O(5)-Sm(2)-O(6) 59.2(2)

O(20)-Sm(2)-O(6) 68.8(3)

O(25)-Sm(2)-O(6) 130.5(2)

O(7)-Zn(1)-O(2) 117.7(3)

O(7)-Zn(1)-O(9) 112.6(3)

O(2)-Zn(1)-O(9) 104.5(3)

O(7)-Zn(1)-N(1) 109.2(3)

O(2)-Zn(1)-N(1) 95.3(3)

O(9)-Zn(1)-N(1) 116.7(3)

O(19)-Zn(2)-O(26) 114.2(3)

O(19)-Zn(2)-O(16) 106.5(4)

O(26)-Zn(2)-O(16) 114.0(3)

O(19)-Zn(2)-O(12) 105.6(3)

O(26)-Zn(2)-O(12) 104.1(3)

O(16)-Zn(2)-O(12) 112.3(3)

O(25)-Zn(3)-O(14) 110.2(3)

O(25)-Zn(3)-O(17) 119.4(3)

O(14)-Zn(3)-O(17) 109.9(3)

O(25)-Zn(3)-O(23) 109.3(3)

O(14)-Zn(3)-O(23) 106.8(3)

O(17)-Zn(3)-O(23) 100.2(3)

O(21)-Zn(4)-O(8) 117.9(3)

O(21)-Zn(4)-N(2) 124.9(3)

O(8)-Zn(4)-N(2) 104.7(3)

O(21)-Zn(4)-O(5) 102.2(3)

O(8)-Zn(4)-O(5) 109.3(3)

N(2)-Zn(4)-O(5) 94.2(3)

**Table S5**. Selected Bond Lengths (Å) and Angles (°) for **4**.

Nd(1)-O(10) 2.255(10)

Nd(1)-O(5) 2.284(10)

Nd(1)-O(2) 2.332(10)

Nd(1)-O(9) 2.34(2)

Nd(1)-O(8) 2.406(13)

Nd(1)-O(1) 2.425(13)

Nd(1)-O(6) 2.469(11)

Nd(1)-O(7) 2.48(2)

Cd(1)-O(5)#1 2.249(10)

Cd(1)-N(1) 2.293(12)

Cd(1)-O(2) 2.298(10)

Cd(1)-O(10) 2.300(9)

Cd(1)-N(2) 2.360(11)

Cd(1)-O(10)#1 2.419(9)

O(10)-Nd(1)-O(5) 76.1(3)

O(10)-Nd(1)-O(2) 73.0(4)

O(5)-Nd(1)-O(2) 89.4(4)

O(10)-Nd(1)-O(9) 78.3(6)

O(5)-Nd(1)-O(9) 92.5(5)

O(2)-Nd(1)-O(9) 149.9(6)

O(10)-Nd(1)-O(8) 135.1(4)

O(5)-Nd(1)-O(8) 142.5(4)

O(2)-Nd(1)-O(8) 116.5(4)

O(9)-Nd(1)-O(8) 78.4(6)

O(10)-Nd(1)-O(1) 134.8(4)

O(5)-Nd(1)-O(1) 89.4(4)

O(2)-Nd(1)-O(1) 64.1(4)

O(9)-Nd(1)-O(1) 145.9(6)

O(8)-Nd(1)-O(1) 79.6(5)

O(10)-Nd(1)-O(6) 132.9(4)

O(5)-Nd(1)-O(6) 66.9(3)

O(2)-Nd(1)-O(6) 132.1(4)

O(9)-Nd(1)-O(6) 75.3(6)

O(8)-Nd(1)-O(6) 75.6(4)

O(1)-Nd(1)-O(6) 74.2(4)

O(10)-Nd(1)-O(7) 93.7(6)

O(5)-Nd(1)-O(7) 169.4(6)

O(2)-Nd(1)-O(7) 90.5(6)

O(9)-Nd(1)-O(7) 82.4(7)

O(8)-Nd(1)-O(7) 45.5(7)

O(1)-Nd(1)-O(7) 100.1(6)

O(6)-Nd(1)-O(7) 120.2(6)

O(5)#1-Cd(1)-N(1) 96.2(4)

O(5)#1-Cd(1)-O(2) 167.4(3)

N(1)-Cd(1)-O(2) 82.5(4)

O(5)#1-Cd(1)-O(10) 106.5(4)

N(1)-Cd(1)-O(10) 154.5(4)

O(2)-Cd(1)-O(10) 72.8(4)

O(5)#1-Cd(1)-N(2) 80.2(4)

N(1)-Cd(1)-N(2) 99.8(4)

O(2)-Cd(1)-N(2) 112.3(4)

O(10)-Cd(1)-N(2) 95.6(4)

O(5)#1-Cd(1)-O(10)#1 73.5(3)

N(1)-Cd(1)-O(10)#1 94.0(4)

O(2)-Cd(1)-O(10)#1 94.1(3)

O(10)-Cd(1)-O(10)#1 81.6(4)

N(2)-Cd(1)-O(10)#1 151.5(4)

**Table S6**. Selected Bond Lengths (Å) and Angles (°) for **5**.

Yb(1)-O(5) 2.276(7)

Yb(1)-O(10) 2.285(8)

Yb(1)-O(2) 2.317(8)

Yb(1)-O(7) 2.354(12)

Yb(1)-O(9) 2.373(15)

Yb(1)-O(8) 2.443(11)

Yb(1)-O(6) 2.489(9)

Yb(1)-O(1) 2.496(9)

Cd(1)-N(1) 2.276(10)

Cd(1)-O(5)#1 2.298(8)

Cd(1)-O(10) 2.303(7)

Cd(1)-O(2) 2.314(8)

Cd(1)-N(2) 2.331(9)

Cd(1)-O(10)#1 2.392(7)

O(5)-Yb(1)-O(10) 77.1(3)

O(5)-Yb(1)-O(2) 89.2(3)

O(10)-Yb(1)-O(2) 73.3(3)

O(5)-Yb(1)-O(7) 164.4(4)

O(10)-Yb(1)-O(7) 87.4(3)

O(2)-Yb(1)-O(7) 88.1(4)

O(5)-Yb(1)-O(9) 92.2(4)

O(10)-Yb(1)-O(9) 77.5(4)

O(2)-Yb(1)-O(9) 149.6(4)

O(7)-Yb(1)-O(9) 82.5(5)

O(5)-Yb(1)-O(8) 141.4(3)

O(10)-Yb(1)-O(8) 137.2(3)

O(2)-Yb(1)-O(8) 114.4(4)

O(7)-Yb(1)-O(8) 52.6(4)

O(9)-Yb(1)-O(8) 82.2(4)

O(5)-Yb(1)-O(6) 66.2(3)

O(10)-Yb(1)-O(6) 133.3(3)

O(2)-Yb(1)-O(6) 131.1(3)

O(7)-Yb(1)-O(6) 125.9(4)

O(9)-Yb(1)-O(6) 76.2(5)

O(8)-Yb(1)-O(6) 75.4(4)

O(5)-Yb(1)-O(1) 88.5(3)

O(10)-Yb(1)-O(1) 135.0(3)

O(2)-Yb(1)-O(1) 64.0(3)

O(7)-Yb(1)-O(1) 104.0(4)

O(9)-Yb(1)-O(1) 146.3(4)

O(8)-Yb(1)-O(1) 76.6(4)

O(6)-Yb(1)-O(1) 73.3(4)

N(1)-Cd(1)-O(5)#1 97.3(3)

N(1)-Cd(1)-O(10) 154.4(3)

O(5)#1-Cd(1)-O(10) 105.4(3)

N(1)-Cd(1)-O(2) 82.3(3)

O(5)#1-Cd(1)-O(2) 167.9(3)

O(10)-Cd(1)-O(2) 73.0(3)

N(1)-Cd(1)-N(2) 99.2(3)

O(5)#1-Cd(1)-N(2) 79.7(3)

O(10)-Cd(1)-N(2) 96.3(3)

O(2)-Cd(1)-N(2) 112.3(3)

N(1)-Cd(1)-O(10)#1 94.1(3)

O(5)#1-Cd(1)-O(10)#1 74.6(3)

O(10)-Cd(1)-O(10)#1 81.1(3)

O(2)-Cd(1)-O(10)#1 93.4(3)

N(2)-Cd(1)-O(10)#1 152.3(3)

**Table S7**. Selected Bond Lengths (Å) and Angles (°) for **6**.

Sm(1)-O(5) 2.288(6)

Sm(1)-O(10) 2.306(7)

Sm(1)-O(2) 2.319(7)

Sm(1)-O(9) 2.332(13)

Sm(1)-O(7) 2.378(14)

Sm(1)-O(8) 2.437(10)

Sm(1)-O(1) 2.479(8)

Sm(1)-O(6) 2.517(8)

Cd(1)-O(10) 2.296(6)

Cd(1)-O(5)#1 2.298(7)

Cd(1)-O(2) 2.305(6)

Cd(1)-N(1) 2.308(8)

Cd(1)-N(2) 2.325(8)

Cd(1)-O(10)#1 2.402(6)

O(5)-Sm(1)-O(10) 76.9(2)

O(5)-Sm(1)-O(2) 89.6(2)

O(10)-Sm(1)-O(2) 73.1(2)

O(5)-Sm(1)-O(9) 92.4(3)

O(10)-Sm(1)-O(9) 78.8(4)

O(2)-Sm(1)-O(9) 150.6(4)

O(5)-Sm(1)-O(7) 165.6(4)

O(10)-Sm(1)-O(7) 88.8(3)

O(2)-Sm(1)-O(7) 87.3(4)

O(9)-Sm(1)-O(7) 83.5(4)

O(5)-Sm(1)-O(8) 140.2(3)

O(10)-Sm(1)-O(8) 137.8(3)

O(2)-Sm(1)-O(8) 115.6(3)

O(9)-Sm(1)-O(8) 80.2(4)

O(7)-Sm(1)-O(8) 52.6(4)

O(5)-Sm(1)-O(1) 88.7(3)

O(10)-Sm(1)-O(1) 135.9(2)

O(2)-Sm(1)-O(1) 65.2(2)

O(9)-Sm(1)-O(1) 144.1(4)

O(7)-Sm(1)-O(1) 102.7(4)

O(8)-Sm(1)-O(1) 76.3(3)

O(5)-Sm(1)-O(6) 65.9(2)

O(10)-Sm(1)-O(6) 132.4(3)

O(2)-Sm(1)-O(6) 132.2(3)

O(9)-Sm(1)-O(6) 74.3(4)

O(7)-Sm(1)-O(6) 125.5(4)

O(8)-Sm(1)-O(6) 74.6(3)

O(1)-Sm(1)-O(6) 73.5(3)

O(10)-Cd(1)-O(5)#1 104.7(2)

O(10)-Cd(1)-O(2) 73.5(2)

O(5)#1-Cd(1)-O(2) 168.2(2)

O(10)-Cd(1)-N(1) 154.3(3)

O(5)#1-Cd(1)-N(1) 97.8(3)

O(2)-Cd(1)-N(1) 81.9(3)

O(10)-Cd(1)-N(2) 94.9(3)

O(5)#1-Cd(1)-N(2) 80.3(3)

O(2)-Cd(1)-N(2) 111.4(3)

N(1)-Cd(1)-N(2) 101.0(3)

O(10)-Cd(1)-O(10)#1 80.4(2)

O(5)#1-Cd(1)-O(10)#1 74.8(2)

O(2)-Cd(1)-O(10)#1 93.4(2)

N(1)-Cd(1)-O(10)#1 93.9(2)

N(2)-Cd(1)-O(10)#1 152.5(3)
