# Supplementary material for: Luminescent Polynuclear Zn- and Cd-Ln Square-Like Nanoclusters With a Flexible Long-Chain Schiff Base Ligand
Source: Front Chem. 2018 Jul 31;6:321. doi: 10.3389/fchem.2018.00321 (PMC6080591; doi:10.3389/fchem.2018.00321)
Supplement: Supplementary file 3 [file Data_Sheet_2.DOC]

# 1. checkCIF (basic structural check) running for cluster-1

*Checking for embedded fcf data in CIF ...*
*No extractable fcf data in found in CIF*

**checkCIF/PLATON (basic structural check)**

You have not supplied any structure factors. As a result the full set of tests cannot be run.

THIS REPORT IS FOR GUIDANCE ONLY. IF USED AS PART OF A REVIEW PROCEDURE FOR PUBLICATION, IT SHOULD NOT REPLACE THE EXPERTISE OF AN EXPERIENCED CRYSTALLOGRAPHIC REFEREE.

You have not supplied any structure factors. As a result the full set of tests cannot be run.

No syntax errors found. [CIF dictionary](http://www.iucr.org/iucr-top/cif/cif_core/definitions/index.html)
Please wait while processing .... [Interpreting this report](http://journals.iucr.org/services/cif/checking/checkcifreport.html)

**Datablock: cluster-1**

| Bond precision: | C-C = 0.0140 A | Wavelength=0.71073 |
| --- | --- | --- |

| Cell: | a=12.077(4) | b=26.643(9) | c=20.099(7) |
| --- | --- | --- | --- |
|  | alpha=90 | beta=95.486(6) | gamma=90 |
| Temperature: | 190 K |  |  |

|  | Calculated | Reported |
| --- | --- | --- |
| Volume | 6438(4) | 6438(4) |
| Space group | P 21/c | P2(1)/c |
| Hall group | -P 2ybc | ? |
| Moiety formula | C84 H116 N4 Nd4 O56 Zn8, 2(C2 H6 O), 2(O) | ? |
| Sum formula | C88 H128 N4 Nd4 O60 Zn8 | C88 H128 N4 Nd4 O60 Zn8 |
| Mr | 3302.03 | 3301.86 |
| Dx,g cm-3 | 1.703 | 1.703 |
| Z | 2 | 2 |
| Mu (mm-1) | 3.131 | 3.132 |
| F000 | 3288.0 | 3288.0 |
| F000' | 3292.57 |  |
| h,k,lmax | 14,31,23 | 14,31,23 |
| Nref | 11333 | 11280 |
| Tmin,Tmax | 0.557,0.709 | 0.652,1.000 |
| Tmin' | 0.546 |  |

| Correction method= # Reported T Limits: Tmin=0.652 Tmax=1.000 AbsCorr = MULTI-SCAN |  |
| --- | --- |

| Data completeness= 0.995 | Theta(max)= 25.000 |
| --- | --- |

| R(reflections)= 0.0417( 8123) | wR2(reflections)= 0.1385( 11280) |
| --- | --- |

| S = 1.069 | Npar= 739 |
| --- | --- |

The following ALERTS were generated. Each ALERT has the format

**test-name_ALERT_alert-type_alert-level**.

Click on the hyperlinks for more details of the test.


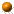
**Alert level B**

[PLAT306_ALERT_2_B](javascript:makeHelpWindow("PLAT306.html")) Isolated Oxygen Atom (H-atoms Missing ?) ....... O1W Check


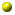
**Alert level C**

[PLAT222_ALERT_3_C](javascript:makeHelpWindow("PLAT222.html")) Non-Solvent Resd 1 H Uiso(max)/Uiso(min) Range 4.2 Ratio

[PLAT234_ALERT_4_C](javascript:makeHelpWindow("PLAT234.html")) Large Hirshfeld Difference C33 -- C34 .. 0.16 Ang.

[PLAT241_ALERT_2_C](javascript:makeHelpWindow("PLAT241.html")) High 'MainMol' Ueq as Compared to Neighbors of O12 Check

**And 7 other PLAT241 Alerts**

More ...

[PLAT242_ALERT_2_C](javascript:makeHelpWindow("PLAT242.html")) Low 'MainMol' Ueq as Compared to Neighbors of Nd2 Check

**And 5 other PLAT242 Alerts**

More ...

[PLAT342_ALERT_3_C](javascript:makeHelpWindow("PLAT342.html")) Low Bond Precision on C-C Bonds ............... 0.01396 Ang.


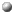
**Alert level G**

[PLAT002_ALERT_2_G](javascript:makeHelpWindow("PLAT002.html")) Number of Distance or Angle Restraints on AtSite 3 Note

[PLAT003_ALERT_2_G](javascript:makeHelpWindow("PLAT003.html")) Number of Uiso or Uij Restrained non-H Atoms ... 82 Report

[PLAT005_ALERT_5_G](javascript:makeHelpWindow("PLAT005.html")) No Embedded Refinement Details found in the CIF Please Do !

[PLAT007_ALERT_5_G](javascript:makeHelpWindow("PLAT007.html")) Number of Unrefined Donor-H Atoms .............. 3 Report

[PLAT083_ALERT_2_G](javascript:makeHelpWindow("PLAT083.html")) SHELXL Second Parameter in WGHT Unusually Large 16.42 Why ?

[PLAT380_ALERT_4_G](javascript:makeHelpWindow("PLAT380.html")) Incorrectly? Oriented X(sp2)-Methyl Moiety ..... C23 Check

**And 5 other PLAT380 Alerts**

More ...

[PLAT860_ALERT_3_G](javascript:makeHelpWindow("PLAT860.html")) Number of Least-Squares Restraints ............. 494 Note

[PLAT899_ALERT_4_G](javascript:makeHelpWindow("PLAT899.html")) SHELXL97 is Deprecated and Succeeded by SHELXL 2014 Note

**PLATON version of 27/03/2017; check.def file version of 24/03/2017**

| **Datablock cluster-1** - ellipsoid plot |
| --- |
| 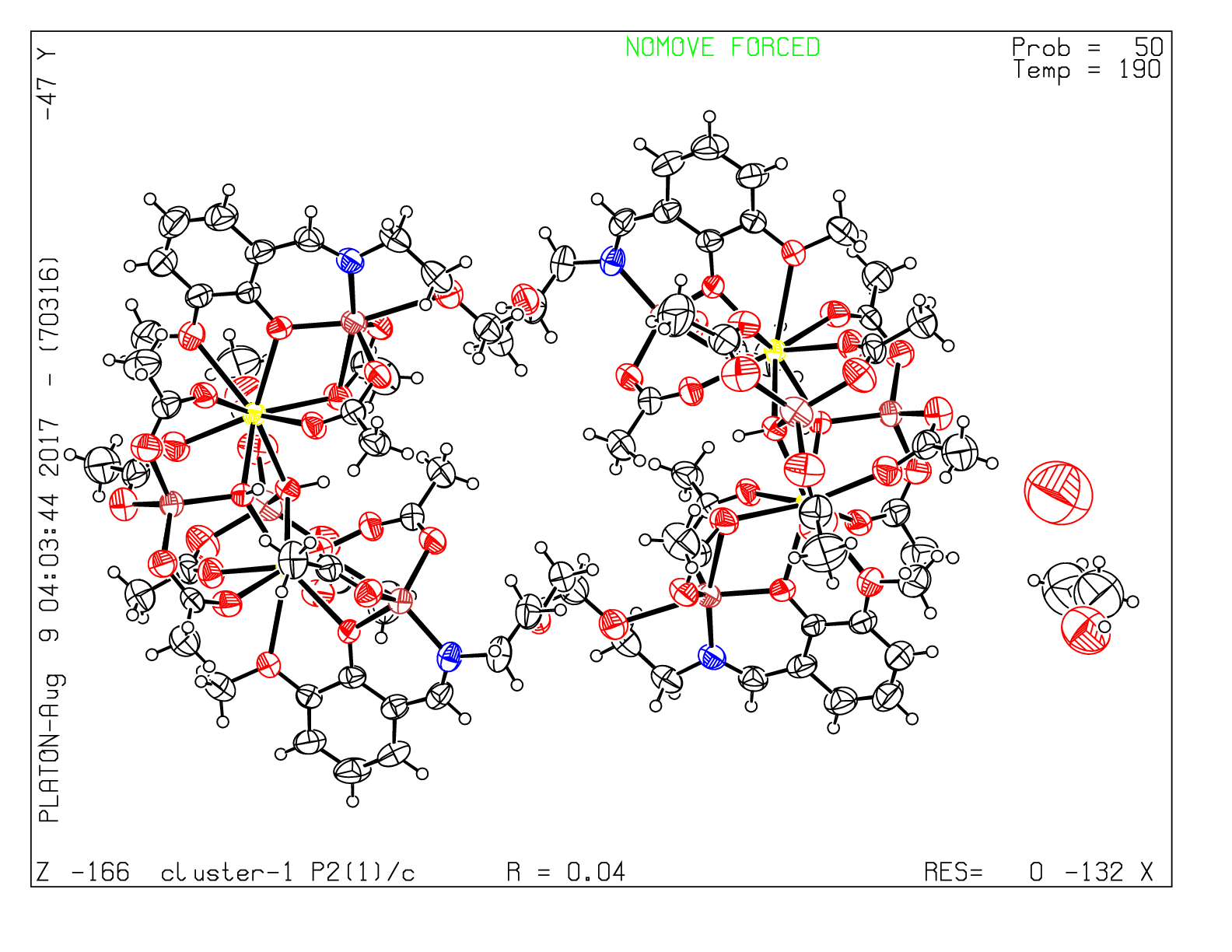 |

# 2. checkCIF (basic structural check) running for cluster-2

*Checking for embedded fcf data in CIF ...*
*No extractable fcf data in found in CIF*

**checkCIF/PLATON (basic structural check)**

You have not supplied any structure factors. As a result the full set of tests cannot be run.

THIS REPORT IS FOR GUIDANCE ONLY. IF USED AS PART OF A REVIEW PROCEDURE FOR PUBLICATION, IT SHOULD NOT REPLACE THE EXPERTISE OF AN EXPERIENCED CRYSTALLOGRAPHIC REFEREE.

You have not supplied any structure factors. As a result the full set of tests cannot be run.

No syntax errors found. [CIF dictionary](http://www.iucr.org/iucr-top/cif/cif_core/definitions/index.html)
Please wait while processing .... [Interpreting this report](http://journals.iucr.org/services/cif/checking/checkcifreport.html)

**Datablock: cluster-2**

| Bond precision: | C-C = 0.0346 A | Wavelength=0.71073 |
| --- | --- | --- |

| Cell: | a=12.0804(4) | b=26.5935(8) | c=20.1084(5) |
| --- | --- | --- | --- |
|  | alpha=90 | beta=95.477(2) | gamma=90 |
| Temperature: | 190 K |  |  |

|  | Calculated | Reported |
| --- | --- | --- |
| Volume | 6430.5(3) | 6430.5(3) |
| Space group | P 21/c | P2(1)/c |
| Hall group | -P 2ybc | ? |
| Moiety formula | C84 H116 N4 O56 Yb4 Zn8, 2(C2 H6 O), 2(O) | ? |
| Sum formula | C88 H128 N4 O60 Yb4 Zn8 | C88 H128 N4 O60 Yb4 Zn8 |
| Mr | 3417.23 | 3417.06 |
| Dx,g cm-3 | 1.765 | 1.765 |
| Z | 2 | 2 |
| Mu (mm-1) | 4.429 | 4.429 |
| F000 | 3368.0 | 3368.0 |
| F000' | 3371.26 |  |
| h,k,lmax | 14,31,23 | 14,30,23 |
| Nref | 11332 | 11018 |
| Tmin,Tmax | 0.594,0.671 | 0.538,1.000 |
| Tmin' | 0.487 |  |

| Correction method= # Reported T Limits: Tmin=0.538 Tmax=1.000 AbsCorr = MULTI-SCAN |  |
| --- | --- |

| Data completeness= 0.972 | Theta(max)= 25.000 |
| --- | --- |

| R(reflections)= 0.1176( 5338) | wR2(reflections)= 0.3495( 11018) |
| --- | --- |

| S = 1.094 | Npar= 739 |
| --- | --- |

The following ALERTS were generated. Each ALERT has the format

**test-name_ALERT_alert-type_alert-level**.

Click on the hyperlinks for more details of the test.


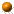
**Alert level B**

[PLAT306_ALERT_2_B](javascript:makeHelpWindow("PLAT306.html")) Isolated Oxygen Atom (H-atoms Missing ?) ....... O1W Check

[PLAT342_ALERT_3_B](javascript:makeHelpWindow("PLAT342.html")) Low Bond Precision on C-C Bonds ............... 0.03464 Ang.


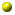
**Alert level C**

[GOODF01_ALERT_2_C](javascript:makeHelpWindow("GOODF_01.html")) The least squares goodness of fit parameter lies

outside the range 0.80 <> 2.00

Goodness of fit given = 2.094

[PLAT026_ALERT_3_C](javascript:makeHelpWindow("PLAT026.html")) Ratio Observed / Unique Reflections (too) Low .. 48 %

[PLAT029_ALERT_3_C](javascript:makeHelpWindow("PLAT029.html")) _diffrn_measured_fraction_theta_full value Low . 0.972 Note

[PLAT082_ALERT_2_C](javascript:makeHelpWindow("PLAT082.html")) High R1 Value .................................. 0.12 Report

[PLAT084_ALERT_3_C](javascript:makeHelpWindow("PLAT084.html")) High wR2 Value (i.e. > 0.25) ................... 0.35 Report

[PLAT213_ALERT_2_C](javascript:makeHelpWindow("PLAT213.html")) Atom C21 has ADP max/min Ratio ..... 3.4 oblate

[PLAT232_ALERT_2_C](javascript:makeHelpWindow("PLAT232.html")) Hirshfeld Test Diff (M-X) Yb1 -- O27 .. 6.7 s.u.

[PLAT232_ALERT_2_C](javascript:makeHelpWindow("PLAT232.html")) Hirshfeld Test Diff (M-X) Zn1 -- O2 .. 6.3 s.u.

[PLAT234_ALERT_4_C](javascript:makeHelpWindow("PLAT234.html")) Large Hirshfeld Difference Yb1 -- O13 .. 0.17 Ang.

**And 12 other PLAT234 Alerts**

More ...

[PLAT241_ALERT_2_C](javascript:makeHelpWindow("PLAT241.html")) High 'MainMol' Ueq as Compared to Neighbors of Yb1 Check

[PLAT241_ALERT_2_C](javascript:makeHelpWindow("PLAT241.html")) High 'MainMol' Ueq as Compared to Neighbors of C18 Check

[PLAT242_ALERT_2_C](javascript:makeHelpWindow("PLAT242.html")) Low 'MainMol' Ueq as Compared to Neighbors of O2 Check

**And 6 other PLAT242 Alerts**

More ...

[PLAT244_ALERT_4_C](javascript:makeHelpWindow("PLAT244.html")) Low 'Solvent' Ueq as Compared to Neighbors of C44 Check

[PLAT410_ALERT_2_C](javascript:makeHelpWindow("PLAT410.html")) Short Intra H...H Contact H8A .. H9B . 1.98 Ang.


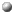
**Alert level G**

[PLAT002_ALERT_2_G](javascript:makeHelpWindow("PLAT002.html")) Number of Distance or Angle Restraints on AtSite 5 Note

[PLAT003_ALERT_2_G](javascript:makeHelpWindow("PLAT003.html")) Number of Uiso or Uij Restrained non-H Atoms ... 82 Report

[PLAT005_ALERT_5_G](javascript:makeHelpWindow("PLAT005.html")) No Embedded Refinement Details found in the CIF Please Do !

[PLAT007_ALERT_5_G](javascript:makeHelpWindow("PLAT007.html")) Number of Unrefined Donor-H Atoms .............. 3 Report

[PLAT083_ALERT_2_G](javascript:makeHelpWindow("PLAT083.html")) SHELXL Second Parameter in WGHT Unusually Large 16.42 Why ?

[PLAT721_ALERT_1_G](javascript:makeHelpWindow("PLAT721.html")) Bond Calc 0.97000, Rep 0.96000 Dev... 0.01 Ang.

C37 -H37B 1.555 1.555 ............ # 150 Check

[PLAT774_ALERT_1_G](javascript:makeHelpWindow("PLAT774.html")) Suspect X-Y Bond in CIF: Yb1 -- Yb2 .. 4.21 Ang.

[PLAT860_ALERT_3_G](javascript:makeHelpWindow("PLAT860.html")) Number of Least-Squares Restraints ............. 495 Note

[PLAT899_ALERT_4_G](javascript:makeHelpWindow("PLAT899.html")) SHELXL97 is Deprecated and Succeeded by SHELXL 2014 Note

**PLATON version of 27/03/2017; check.def file version of 24/03/2017**

| **Datablock cluster-2** - ellipsoid plot |
| --- |
| 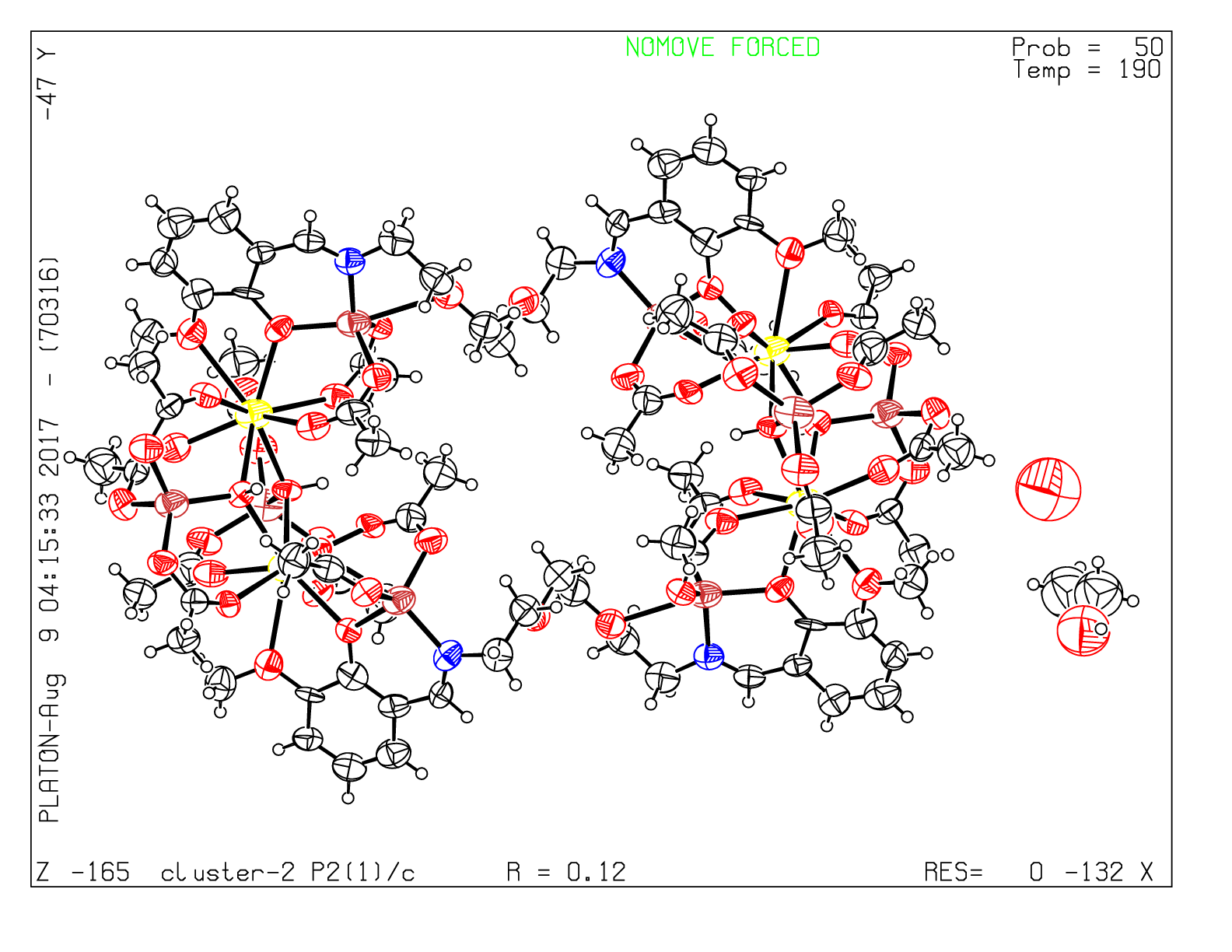 |

# 3. checkCIF (basic structural check) running for cluster-3

*Checking for embedded fcf data in CIF ...*
*No extractable fcf data in found in CIF*

**checkCIF/PLATON (basic structural check)**

You have not supplied any structure factors. As a result the full set of tests cannot be run.

THIS REPORT IS FOR GUIDANCE ONLY. IF USED AS PART OF A REVIEW PROCEDURE FOR PUBLICATION, IT SHOULD NOT REPLACE THE EXPERTISE OF AN EXPERIENCED CRYSTALLOGRAPHIC REFEREE.

You have not supplied any structure factors. As a result the full set of tests cannot be run.

No syntax errors found. [CIF dictionary](http://www.iucr.org/iucr-top/cif/cif_core/definitions/index.html)
Please wait while processing .... [Interpreting this report](http://journals.iucr.org/services/cif/checking/checkcifreport.html)

**Datablock: cluster-3**

| Bond precision: | C-C = 0.0164 A | Wavelength=0.71073 |
| --- | --- | --- |

| Cell: | a=11.929(2) | b=26.173(5) | c=20.016(3) |
| --- | --- | --- | --- |
|  | alpha=90 | beta=95.430(4) | gamma=90 |
| Temperature: | 190 K |  |  |

|  | Calculated | Reported |
| --- | --- | --- |
| Volume | 6221.3(18) | 6221.3(19) |
| Space group | P 21/c | P2(1)/c |
| Hall group | -P 2ybc | ? |
| Moiety formula | C84 H116 N4 O56 Sm4 Zn8, 2(C2 H6 O), 2(O) | ? |
| Sum formula | C88 H128 N4 O60 Sm4 Zn8 | C88 H128 N4 O60 Sm4 Zn8 |
| Mr | 3326.51 | 3326.30 |
| Dx,g cm-3 | 1.776 | 1.776 |
| Z | 2 | 2 |
| Mu (mm-1) | 3.459 | 3.459 |
| F000 | 3304.0 | 3304.0 |
| F000' | 3308.83 |  |
| h,k,lmax | 14,31,23 | 14,31,23 |
| Nref | 10957 | 10913 |
| Tmin,Tmax | 0.542,0.638 | 0.611,1.000 |
| Tmin' | 0.463 |  |

| Correction method= # Reported T Limits: Tmin=0.611 Tmax=1.000 AbsCorr = MULTI-SCAN |  |
| --- | --- |

| Data completeness= 0.996 | Theta(max)= 25.000 |
| --- | --- |

| R(reflections)= 0.0567( 6984) | wR2(reflections)= 0.1703( 10913) |
| --- | --- |

| S = 1.036 | Npar= 739 |
| --- | --- |

The following ALERTS were generated. Each ALERT has the format

**test-name_ALERT_alert-type_alert-level**.

Click on the hyperlinks for more details of the test.


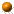
**Alert level B**

[PLAT094_ALERT_2_B](javascript:makeHelpWindow("PLAT094.html")) Ratio of Maximum / Minimum Residual Density .... 5.24 Report

[PLAT306_ALERT_2_B](javascript:makeHelpWindow("PLAT306.html")) Isolated Oxygen Atom (H-atoms Missing ?) ....... O1W Check


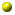
**Alert level C**

[PLAT220_ALERT_2_C](javascript:makeHelpWindow("PLAT220.html")) Non-Solvent Resd 1 C Ueq(max)/Ueq(min) Range 3.1 Ratio

[PLAT342_ALERT_3_C](javascript:makeHelpWindow("PLAT342.html")) Low Bond Precision on C-C Bonds ............... 0.01639 Ang.


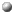
**Alert level G**

[PLAT002_ALERT_2_G](javascript:makeHelpWindow("PLAT002.html")) Number of Distance or Angle Restraints on AtSite 3 Note

[PLAT003_ALERT_2_G](javascript:makeHelpWindow("PLAT003.html")) Number of Uiso or Uij Restrained non-H Atoms ... 82 Report

[PLAT005_ALERT_5_G](javascript:makeHelpWindow("PLAT005.html")) No Embedded Refinement Details found in the CIF Please Do !

[PLAT007_ALERT_5_G](javascript:makeHelpWindow("PLAT007.html")) Number of Unrefined Donor-H Atoms .............. 3 Report

[PLAT083_ALERT_2_G](javascript:makeHelpWindow("PLAT083.html")) SHELXL Second Parameter in WGHT Unusually Large 59.25 Why ?

[PLAT380_ALERT_4_G](javascript:makeHelpWindow("PLAT380.html")) Incorrectly? Oriented X(sp2)-Methyl Moiety ..... C33 Check

[PLAT860_ALERT_3_G](javascript:makeHelpWindow("PLAT860.html")) Number of Least-Squares Restraints ............. 494 Note

[PLAT899_ALERT_4_G](javascript:makeHelpWindow("PLAT899.html")) SHELXL97 is Deprecated and Succeeded by SHELXL 2014 Note

**PLATON version of 27/03/2017; check.def file version of 24/03/2017**

| **Datablock cluster-3** - ellipsoid plot |
| --- |
| 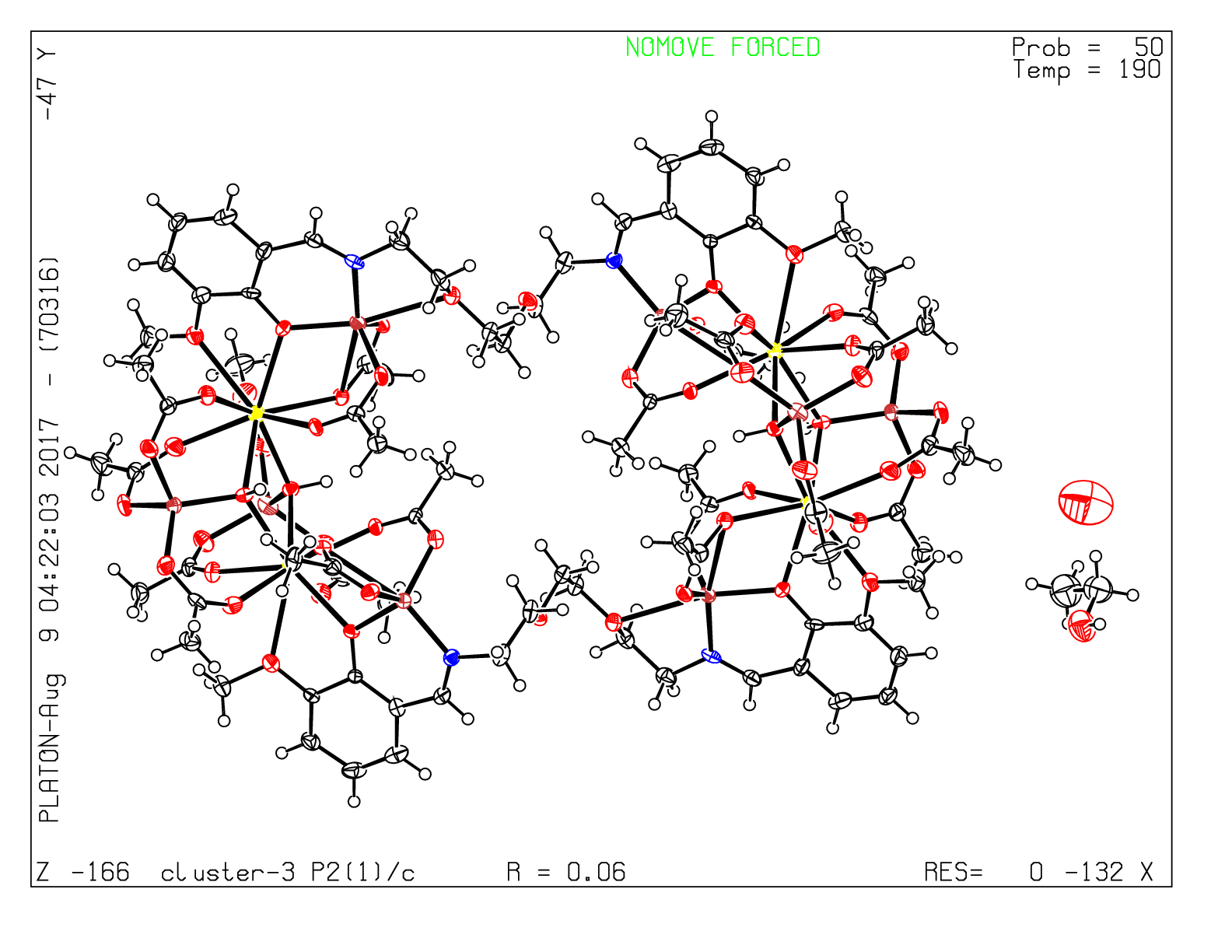 |

# 4. checkCIF (basic structural check) running for cluster-4

*Checking for embedded fcf data in CIF ...*
*No extractable fcf data in found in CIF*

**checkCIF/PLATON (basic structural check)**

You have not supplied any structure factors. As a result the full set of tests cannot be run.

THIS REPORT IS FOR GUIDANCE ONLY. IF USED AS PART OF A REVIEW PROCEDURE FOR PUBLICATION, IT SHOULD NOT REPLACE THE EXPERTISE OF AN EXPERIENCED CRYSTALLOGRAPHIC REFEREE.

You have not supplied any structure factors. As a result the full set of tests cannot be run.

No syntax errors found. [CIF dictionary](http://www.iucr.org/iucr-top/cif/cif_core/definitions/index.html)
Please wait while processing .... [Interpreting this report](http://journals.iucr.org/services/cif/checking/checkcifreport.html)

**Datablock: y**

| Bond precision: | C-C = 0.0233 A | Wavelength=0.71073 |
| --- | --- | --- |

| Cell: | a=12.377(4) | b=16.508(5) | c=14.290(5) |
| --- | --- | --- | --- |
|  | alpha=90 | beta=95.849(6) | gamma=90 |
| Temperature: | 190 K |  |  |

|  | Calculated | Reported |
| --- | --- | --- |
| Volume | 2904.5(16) | 2904.6(17) |
| Space group | P 21/n | P2(1)/n |
| Hall group | -P 2yn | ? |
| Moiety formula | C50 H66 Cd2 N4 Nd2 O20 | ? |
| Sum formula | C50 H66 Cd2 N4 Nd2 O20 | C50 H66 Cd2 N4 Nd2 O20 |
| Mr | 1556.37 | 1556.35 |
| Dx,g cm-3 | 1.780 | 1.780 |
| Z | 2 | 2 |
| Mu (mm-1) | 2.555 | 2.555 |
| F000 | 1540.0 | 1540.0 |
| F000' | 1536.65 |  |
| h,k,lmax | 14,19,16 | 14,19,16 |
| Nref | 5114 | 4885 |
| Tmin,Tmax | 0.699,0.795 | 0.702,1.000 |
| Tmin' | 0.658 |  |

| Correction method= # Reported T Limits: Tmin=0.702 Tmax=1.000 AbsCorr = MULTI-SCAN |  |
| --- | --- |

| Data completeness= 0.955 | Theta(max)= 25.000 |
| --- | --- |

| R(reflections)= 0.0819( 3704) | wR2(reflections)= 0.3216( 4885) |
| --- | --- |

| S = 1.335 | Npar= 352 |
| --- | --- |

The following ALERTS were generated. Each ALERT has the format

**test-name_ALERT_alert-type_alert-level**.

Click on the hyperlinks for more details of the test.


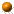
**Alert level B**

[PLAT029_ALERT_3_B](javascript:makeHelpWindow("PLAT029.html")) _diffrn_measured_fraction_theta_full value Low . 0.955 Note

[PLAT230_ALERT_2_B](javascript:makeHelpWindow("PLAT230.html")) Hirshfeld Test Diff for O7 -- C24 .. 8.5 s.u.

[PLAT241_ALERT_2_B](javascript:makeHelpWindow("PLAT241.html")) High 'MainMol' Ueq as Compared to Neighbors of O7 Check

[PLAT342_ALERT_3_B](javascript:makeHelpWindow("PLAT342.html")) Low Bond Precision on C-C Bonds ............... 0.02328 Ang.


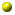
**Alert level C**

[PLAT084_ALERT_3_C](javascript:makeHelpWindow("PLAT084.html")) High wR2 Value (i.e. > 0.25) ................... 0.32 Report

[PLAT220_ALERT_2_C](javascript:makeHelpWindow("PLAT220.html")) Non-Solvent Resd 1 C Ueq(max)/Ueq(min) Range 3.1 Ratio

[PLAT230_ALERT_2_C](javascript:makeHelpWindow("PLAT230.html")) Hirshfeld Test Diff for O6 -- C22 .. 5.1 s.u.

[PLAT230_ALERT_2_C](javascript:makeHelpWindow("PLAT230.html")) Hirshfeld Test Diff for O10 -- C25 .. 6.0 s.u.

[PLAT232_ALERT_2_C](javascript:makeHelpWindow("PLAT232.html")) Hirshfeld Test Diff (M-X) Nd1 -- O7 .. 7.5 s.u.

[PLAT232_ALERT_2_C](javascript:makeHelpWindow("PLAT232.html")) Hirshfeld Test Diff (M-X) Nd1 -- O9 .. 6.6 s.u.

[PLAT234_ALERT_4_C](javascript:makeHelpWindow("PLAT234.html")) Large Hirshfeld Difference O8 -- C24 .. 0.18 Ang.

**And 2 other PLAT234 Alerts**

More ...

[PLAT241_ALERT_2_C](javascript:makeHelpWindow("PLAT241.html")) High 'MainMol' Ueq as Compared to Neighbors of O8 Check

[PLAT241_ALERT_2_C](javascript:makeHelpWindow("PLAT241.html")) High 'MainMol' Ueq as Compared to Neighbors of C19 Check

[PLAT242_ALERT_2_C](javascript:makeHelpWindow("PLAT242.html")) Low 'MainMol' Ueq as Compared to Neighbors of Nd1 Check

**And 2 other PLAT242 Alerts**

More ...

[PLAT410_ALERT_2_C](javascript:makeHelpWindow("PLAT410.html")) Short Intra H...H Contact H8A .. H9C . 1.96 Ang.


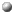
**Alert level G**

[PLAT002_ALERT_2_G](javascript:makeHelpWindow("PLAT002.html")) Number of Distance or Angle Restraints on AtSite 6 Note

[PLAT003_ALERT_2_G](javascript:makeHelpWindow("PLAT003.html")) Number of Uiso or Uij Restrained non-H Atoms ... 39 Report

[PLAT005_ALERT_5_G](javascript:makeHelpWindow("PLAT005.html")) No Embedded Refinement Details found in the CIF Please Do !

[PLAT072_ALERT_2_G](javascript:makeHelpWindow("PLAT072.html")) SHELXL First Parameter in WGHT Unusually Large 0.20 Report

[PLAT335_ALERT_2_G](javascript:makeHelpWindow("PLAT335.html")) Check Large C6 Ring C-C Range C2 -C7 0.16 Ang.

[PLAT343_ALERT_2_G](javascript:makeHelpWindow("PLAT343.html")) Unusual sp3 Angle Range in Main Residue for C15 Check

[PLAT721_ALERT_1_G](javascript:makeHelpWindow("PLAT721.html")) Bond Calc 0.97000, Rep 0.96000 Dev... 0.01 Ang.

C1 -H1B 1.555 1.555 ............ # 39 Check

[PLAT721_ALERT_1_G](javascript:makeHelpWindow("PLAT721.html")) Bond Calc 0.97000, Rep 0.96000 Dev... 0.01 Ang.

C22 -H22C 1.555 1.555 ............ # 82 Check

[PLAT764_ALERT_4_G](javascript:makeHelpWindow("PLAT764.html")) Overcomplete CIF Bond List Detected (Rep/Expd) . 1.14 Ratio

[PLAT860_ALERT_3_G](javascript:makeHelpWindow("PLAT860.html")) Number of Least-Squares Restraints ............. 237 Note

[PLAT899_ALERT_4_G](javascript:makeHelpWindow("PLAT899.html")) SHELXL97 is Deprecated and Succeeded by SHELXL 2014 Note

**PLATON version of 27/03/2017; check.def file version of 24/03/2017**

| **Datablock y** - ellipsoid plot |
| --- |
| 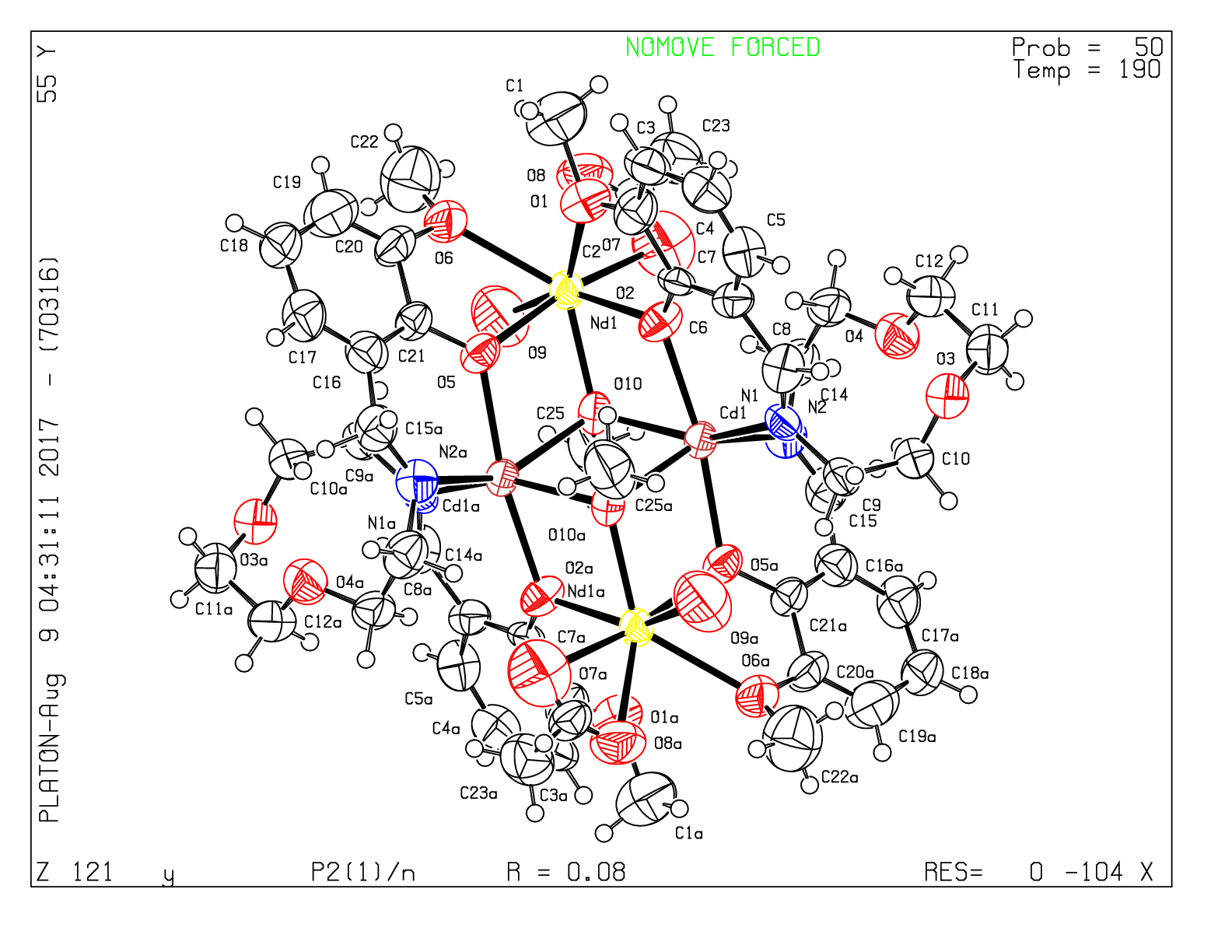 |

# 5. checkCIF/PLATON (basic structural check) for cluster-5

You have not supplied any structure factors. As a result the full set of tests cannot be run.

THIS REPORT IS FOR GUIDANCE ONLY. IF USED AS PART OF A REVIEW PROCEDURE FOR PUBLICATION, IT SHOULD NOT REPLACE THE EXPERTISE OF AN EXPERIENCED CRYSTALLOGRAPHIC REFEREE.

You have not supplied any structure factors. As a result the full set of tests cannot be run.

No syntax errors found. [CIF dictionary](http://www.iucr.org/iucr-top/cif/cif_core/definitions/index.html)
Please wait while processing .... [Interpreting this report](http://journals.iucr.org/services/cif/checking/checkcifreport.html)

**Datablock: cluster-5**

| Bond precision: | C-C = 0.0183 A | Wavelength=0.71073 |
| --- | --- | --- |

| Cell: | a=12.4151(6) | b=16.5360(8) | c=14.3093(7) |
| --- | --- | --- | --- |
|  | alpha=90 | beta=96.345(1) | gamma=90 |
| Temperature: | 190 K |  |  |

|  | Calculated | Reported |
| --- | --- | --- |
| Volume | 2919.7(2) | 2919.6(2) |
| Space group | P 21/n | P2(1)/n |
| Hall group | -P 2yn | ? |
| Moiety formula | C50 H66 Cd2 N4 O20 Yb2 | ? |
| Sum formula | C50 H66 Cd2 N4 O20 Yb2 | C50 H66 Cd2 N4 O20 Yb2 |
| Mr | 1613.97 | 1613.95 |
| Dx,g cm-3 | 1.836 | 1.836 |
| Z | 2 | 2 |
| Mu (mm-1) | 3.966 | 3.967 |
| F000 | 1580.0 | 1580.0 |
| F000' | 1575.97 |  |
| h,k,lmax | 14,19,17 | 14,19,17 |
| Nref | 5133 | 5119 |
| Tmin,Tmax | 0.495,0.728 | 0.579,1.000 |
| Tmin' | 0.466 |  |

| Correction method= # Reported T Limits: Tmin=0.579 Tmax=1.000 AbsCorr = MULTI-SCAN |  |
| --- | --- |

| Data completeness= 0.997 | Theta(max)= 25.000 |
| --- | --- |

| R(reflections)= 0.0639( 4053) | wR2(reflections)= 0.2571( 5119) |
| --- | --- |

| S = 1.091 | Npar= 352 |
| --- | --- |

The following ALERTS were generated. Each ALERT has the format

**test-name_ALERT_alert-type_alert-level**.

Click on the hyperlinks for more details of the test.


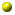
**Alert level C**

[PLAT084_ALERT_3_C](javascript:makeHelpWindow("PLAT084.html")) High wR2 Value (i.e. > 0.25) ................... 0.26 Report

[PLAT220_ALERT_2_C](javascript:makeHelpWindow("PLAT220.html")) Non-Solvent Resd 1 C Ueq(max)/Ueq(min) Range 3.3 Ratio

[PLAT230_ALERT_2_C](javascript:makeHelpWindow("PLAT230.html")) Hirshfeld Test Diff for O10 -- C25 .. 6.2 s.u.

[PLAT234_ALERT_4_C](javascript:makeHelpWindow("PLAT234.html")) Large Hirshfeld Difference Yb1 -- O9 .. 0.17 Ang.

**And 2 other PLAT234 Alerts**

More ...

[PLAT241_ALERT_2_C](javascript:makeHelpWindow("PLAT241.html")) High 'MainMol' Ueq as Compared to Neighbors of O7 Check

[PLAT241_ALERT_2_C](javascript:makeHelpWindow("PLAT241.html")) High 'MainMol' Ueq as Compared to Neighbors of O8 Check

[PLAT242_ALERT_2_C](javascript:makeHelpWindow("PLAT242.html")) Low 'MainMol' Ueq as Compared to Neighbors of O10 Check

[PLAT242_ALERT_2_C](javascript:makeHelpWindow("PLAT242.html")) Low 'MainMol' Ueq as Compared to Neighbors of C24 Check

[PLAT342_ALERT_3_C](javascript:makeHelpWindow("PLAT342.html")) Low Bond Precision on C-C Bonds ............... 0.01828 Ang.


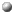
**Alert level G**

[PLAT002_ALERT_2_G](javascript:makeHelpWindow("PLAT002.html")) Number of Distance or Angle Restraints on AtSite 6 Note

[PLAT003_ALERT_2_G](javascript:makeHelpWindow("PLAT003.html")) Number of Uiso or Uij Restrained non-H Atoms ... 39 Report

[PLAT005_ALERT_5_G](javascript:makeHelpWindow("PLAT005.html")) No Embedded Refinement Details found in the CIF Please Do !

[PLAT072_ALERT_2_G](javascript:makeHelpWindow("PLAT072.html")) SHELXL First Parameter in WGHT Unusually Large 0.20 Report

[PLAT343_ALERT_2_G](javascript:makeHelpWindow("PLAT343.html")) Unusual sp3 Angle Range in Main Residue for C15 Check

[PLAT764_ALERT_4_G](javascript:makeHelpWindow("PLAT764.html")) Overcomplete CIF Bond List Detected (Rep/Expd) . 1.16 Ratio

[PLAT779_ALERT_4_G](javascript:makeHelpWindow("PLAT779.html")) Suspect or Irrelevant (Bond) Angle in CIF .... # 210 Check

O5 -C21 -YB1 1.555 1.555 1.555 36.30 Deg.

[PLAT860_ALERT_3_G](javascript:makeHelpWindow("PLAT860.html")) Number of Least-Squares Restraints ............. 237 Note

[PLAT899_ALERT_4_G](javascript:makeHelpWindow("PLAT899.html")) SHELXL97 is Deprecated and Succeeded by SHELXL 2014 Note

**PLATON version of 27/03/2017; check.def file version of 24/03/2017**

| **Datablock cluster-5** - ellipsoid plot |
| --- |
| 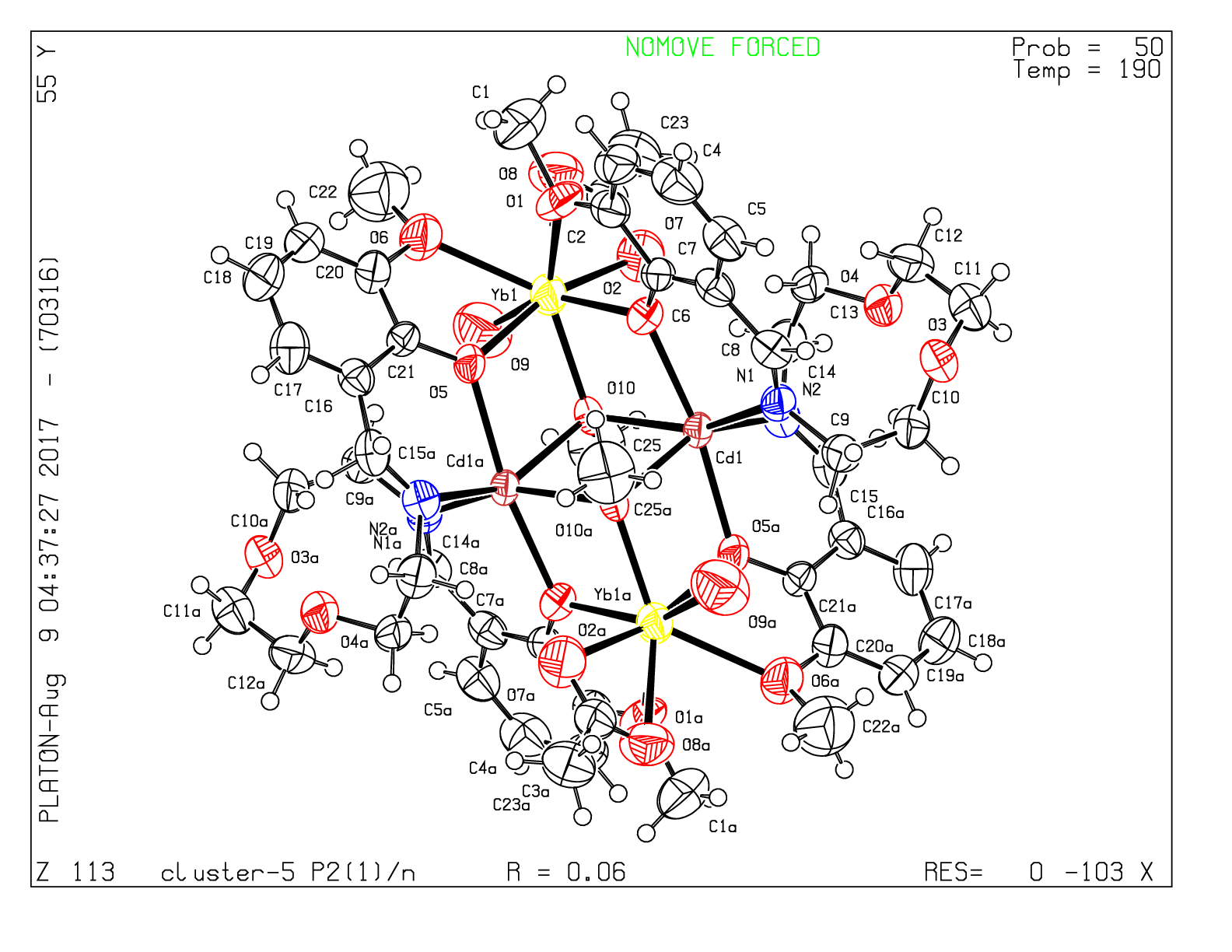 |

# [Download CIF editor (publCIF) from the IUCr](http://journals.iucr.org/services/cif/publcif/)

# 6. checkCIF (basic structural check) running for cluster-6

*Checking for embedded fcf data in CIF ...*
*No extractable fcf data in found in CIF*

**checkCIF/PLATON (basic structural check)**

You have not supplied any structure factors. As a result the full set of tests cannot be run.

THIS REPORT IS FOR GUIDANCE ONLY. IF USED AS PART OF A REVIEW PROCEDURE FOR PUBLICATION, IT SHOULD NOT REPLACE THE EXPERTISE OF AN EXPERIENCED CRYSTALLOGRAPHIC REFEREE.

You have not supplied any structure factors. As a result the full set of tests cannot be run.

No syntax errors found. [CIF dictionary](http://www.iucr.org/iucr-top/cif/cif_core/definitions/index.html)
Please wait while processing .... [Interpreting this report](http://journals.iucr.org/services/cif/checking/checkcifreport.html)

**Datablock: cluster-6**

| Bond precision: | C-C = 0.0159 A | Wavelength=0.71073 |
| --- | --- | --- |

| Cell: | a=12.387(2) | b=16.580(3) | c=14.324(2) |
| --- | --- | --- | --- |
|  | alpha=90 | beta=95.789(4) | gamma=90 |
| Temperature: | 190 K |  |  |

|  | Calculated | Reported |
| --- | --- | --- |
| Volume | 2926.8(8) | 2926.9(8) |
| Space group | P 21/n | P2(1)/n |
| Hall group | -P 2yn | ? |
| Moiety formula | C50 H66 Cd2 N4 O20 Sm2 | ? |
| Sum formula | C50 H66 Cd2 N4 O20 Sm2 | C50 H66 Cd2 N4 O20 Sm2 |
| Mr | 1568.61 | 1568.57 |
| Dx,g cm-3 | 1.780 | 1.780 |
| Z | 2 | 2 |
| Mu (mm-1) | 2.768 | 2.768 |
| F000 | 1548.0 | 1548.0 |
| F000' | 1544.78 |  |
| h,k,lmax | 14,19,17 | 14,19,17 |
| Nref | 5149 | 5133 |
| Tmin,Tmax | 0.742,0.824 | 0.601,1.000 |
| Tmin' | 0.660 |  |

| Correction method= # Reported T Limits: Tmin=0.601 Tmax=1.000 AbsCorr = MULTI-SCAN |  |
| --- | --- |

| Data completeness= 0.997 | Theta(max)= 25.000 |
| --- | --- |

| R(reflections)= 0.0594( 4248) | wR2(reflections)= 0.2425( 5133) |
| --- | --- |

| S = 1.059 | Npar= 352 |
| --- | --- |

The following ALERTS were generated. Each ALERT has the format

**test-name_ALERT_alert-type_alert-level**.

Click on the hyperlinks for more details of the test.


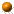
**Alert level B**

[PLAT230_ALERT_2_B](javascript:makeHelpWindow("PLAT230.html")) Hirshfeld Test Diff for O10 -- C25 .. 9.5 s.u.

[PLAT241_ALERT_2_B](javascript:makeHelpWindow("PLAT241.html")) High 'MainMol' Ueq as Compared to Neighbors of O7 Check


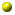
**Alert level C**

[PLAT220_ALERT_2_C](javascript:makeHelpWindow("PLAT220.html")) Non-Solvent Resd 1 C Ueq(max)/Ueq(min) Range 3.4 Ratio

[PLAT230_ALERT_2_C](javascript:makeHelpWindow("PLAT230.html")) Hirshfeld Test Diff for O6 -- C22 .. 5.5 s.u.

[PLAT232_ALERT_2_C](javascript:makeHelpWindow("PLAT232.html")) Hirshfeld Test Diff (M-X) Sm1 -- O7 .. 7.2 s.u.

**And 2 other PLAT232 Alerts**

More ...

[PLAT234_ALERT_4_C](javascript:makeHelpWindow("PLAT234.html")) Large Hirshfeld Difference O1 -- C1 .. 0.17 Ang.

**And 3 other PLAT234 Alerts**

More ...

[PLAT241_ALERT_2_C](javascript:makeHelpWindow("PLAT241.html")) High 'MainMol' Ueq as Compared to Neighbors of O8 Check

[PLAT242_ALERT_2_C](javascript:makeHelpWindow("PLAT242.html")) Low 'MainMol' Ueq as Compared to Neighbors of Sm1 Check

**And 2 other PLAT242 Alerts**

More ...

[PLAT342_ALERT_3_C](javascript:makeHelpWindow("PLAT342.html")) Low Bond Precision on C-C Bonds ............... 0.01594 Ang.

[PLAT362_ALERT_2_C](javascript:makeHelpWindow("PLAT362.html")) Short C(sp3)-C(sp2) Bond C15 - C16_a .. 1.41 Ang.


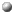
**Alert level G**

[PLAT002_ALERT_2_G](javascript:makeHelpWindow("PLAT002.html")) Number of Distance or Angle Restraints on AtSite 6 Note

[PLAT003_ALERT_2_G](javascript:makeHelpWindow("PLAT003.html")) Number of Uiso or Uij Restrained non-H Atoms ... 39 Report

[PLAT005_ALERT_5_G](javascript:makeHelpWindow("PLAT005.html")) No Embedded Refinement Details found in the CIF Please Do !

[PLAT072_ALERT_2_G](javascript:makeHelpWindow("PLAT072.html")) SHELXL First Parameter in WGHT Unusually Large 0.20 Report

[PLAT343_ALERT_2_G](javascript:makeHelpWindow("PLAT343.html")) Unusual sp3 Angle Range in Main Residue for C15 Check

[PLAT764_ALERT_4_G](javascript:makeHelpWindow("PLAT764.html")) Overcomplete CIF Bond List Detected (Rep/Expd) . 1.14 Ratio

[PLAT860_ALERT_3_G](javascript:makeHelpWindow("PLAT860.html")) Number of Least-Squares Restraints ............. 237 Note

[PLAT899_ALERT_4_G](javascript:makeHelpWindow("PLAT899.html")) SHELXL97 is Deprecated and Succeeded by SHELXL 2014 Note

**PLATON version of 27/03/2017; check.def file version of 24/03/2017**

| **Datablock cluster-6** - ellipsoid plot |
| --- |
| 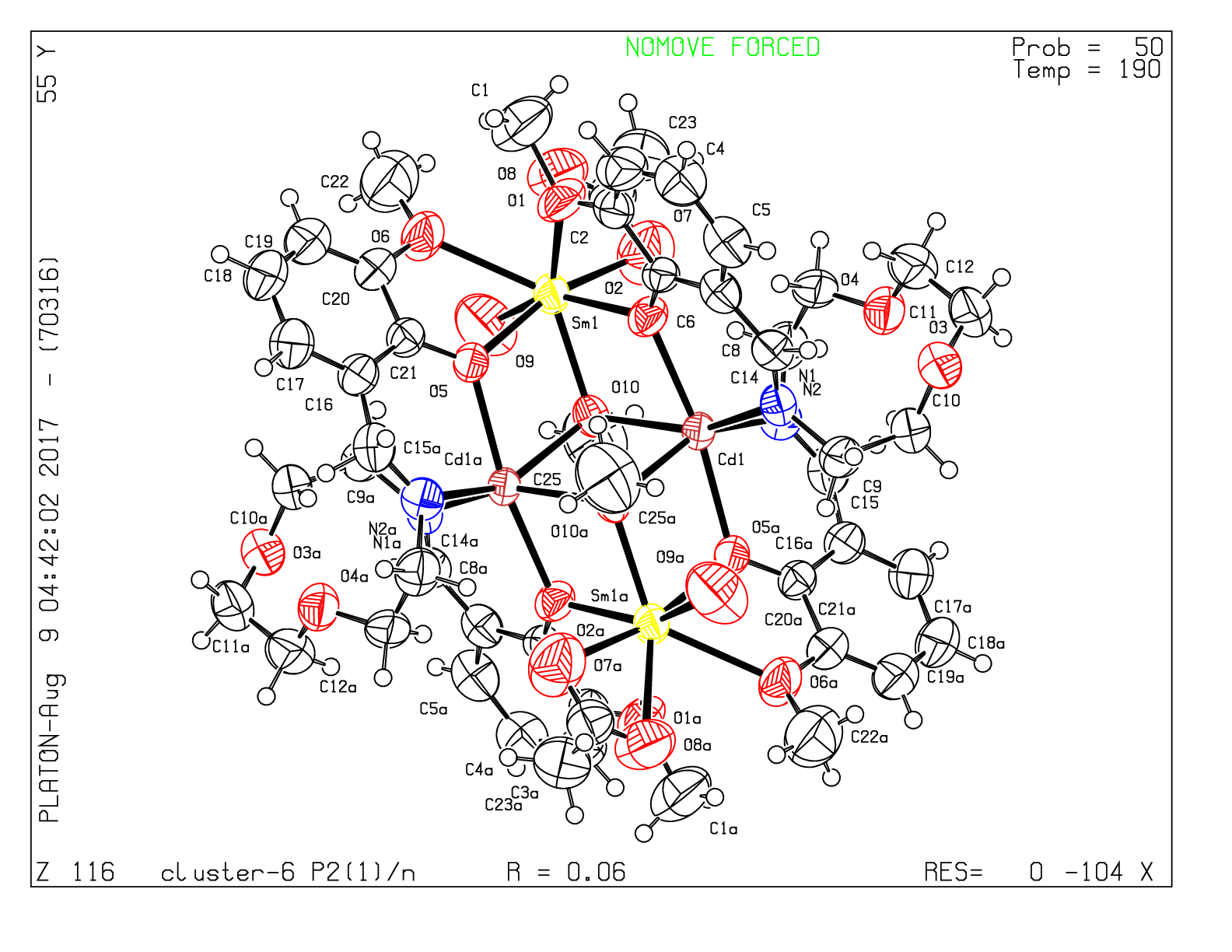 |
